# Cannabinoids in Cancer: Molecular Mechanisms of Tumor Cell Death and Translational Opportunities

**DOI:** 10.3390/biom16091260

**Published:** 2026-08-31

**Authors:** Alaa A. El Moghrabi, Ali Al Khatib, Israa Ahmad Cheikh, Charbel Al Hage, Dima Ismail, Mariam Zhour, Philip Mwesigwa, Nadine Darwiche

**Affiliations:** 1 Department of Biochemistry and Molecular Genetics, American University of Beirut, Beirut 1107-2020, Lebanon; aae116@mail.aub.edu (A.A.E.M.); ana80@mail.aub.edu (A.A.K.); iac06@mail.aub.edu (I.A.C.); cja30@mail.aub.edu (C.A.H.); maz45@mail.aub.edu (M.Z.); pjm09@mail.aub.edu (P.M.); 2 Department of Experimental Pathology, Immunology and Microbiology, American University of Beirut, Beirut 1107-2020, Lebanon; dmi15@mail.aub.edu

**Keywords:** cannabinoids, phytocannabinoids, cancer, cell death, apoptosis, autophagy

## Abstract

Cannabinoids are terpenophenolic compounds derived from *Cannabis sativa* L. that exert a broad range of biological and pharmacological activities. Increasing evidence highlights their potential as modulators of cancer progression specifically through the suppression of tumor cell growth, angiogenesis, and metastasis across multiple tumor models. This review provides a comprehensive overview of the molecular mechanisms by which natural and synthetic cannabinoids induce regulated cancer cell death. Current evidence demonstrates that cannabinoids regulate multiple forms of cancer cell death, including apoptosis, autophagy-dependent cell death, necroptosis, ferroptosis, and parthanatos. These effects are mediated through complex and interconnected signaling pathways such as TRIB3/AKT/mTORC1, PI3K/AKT/mTOR, MAPK/ERK, NF-κB, ERK/JNK/p38-MAPK, and ceramide/Raf1/ERK/ROS. In addition to their direct antitumor effects, cannabinoids can enhance the efficacy of conventional anticancer therapies through the coordinated regulation of complementary cell death pathways. They also provide clinically relevant supportive benefits in palliative care, alleviating chemotherapy-induced nausea, cachexia, and mood or sleep disturbances. Collectively, these findings identify cannabinoids as promising anticancer agents and therapeutic adjuvants, predominantly in the preclinical setting. However, significant challenges remain regarding their safety, optimal dosing, formulation, and clinical efficacy. Further mechanistic studies, rigorous preclinical research, and well-designed clinical trials are required to establish the translation of cannabinoid-based therapies into precision oncology.

## 1. Introduction

Cancer is a major public health problem, with approximately 20 million newly diagnosed cases and 10 million global deaths recorded according to the latest GLOBOCAN cancer statistics [1]. Unfortunately, global cancer incidence is projected to rise to 35 million cases by 2050 [1]. Traditional cancer treatments, such as surgery, chemotherapy, radiotherapy, and immunotherapy, are often hindered by adverse side effects, drug resistance, and tumor recurrence, calling for more efficient therapeutic strategies.

In recent years, herbal medicine has gained significant recognition as a promising approach in cancer management [2]. Among many medicinal herbs, the cannabis plants have attracted significant recognition for their diverse therapeutic potential. The cannabis plant, a member of the Cannabaceae family, has been traditionally utilized for centuries for spiritual and remedial healing [3]. This plant is characterized by its psychoactive and non-psychoactive constituents; however, its widespread use for recreational and psychotic purposes led to its prohibition. Recently, the therapeutic potential of cannabis has sparked renewed research interest with the legalization of its use for medical purposes in the United States, Canada, and European Union countries [4,5]. Cannabis extracts are increasingly indicated for neurological and psychiatric disorders and exhibit antioxidant, anti-inflammatory, and analgesic effects [6,7]. Their therapeutic promise is primarily attributed to cannabinoids, which are terpenophenolic compounds with a 21-carbon backbone [8,9].

Cannabinoids are classified into three categories based on their origin: endocannabinoids, phytocannabinoids, and synthetic cannabinoids [10]. The endocannabinoid system is an evolutionarily conserved animal homeostatic network controlling a variety of physiological properties, including immune and endocrine functions, inflammation, pain perception, and brain activity. The endocannabinoid system consists of three major components: endogenous cannabinoids, cannabinoid receptors, and metabolic enzymes. The endogenous cannabinoids (endocannabinoids) primarily consist of 2-arachidonoylglycerol (2-AG) and arachidonoyl ethanolamide (AEA, also known as anandamide). The cannabinoid receptors include the classical cannabinoid receptors type 1 and 2 (CB1 and CB2) and other non-canonical receptors such as G protein-coupled receptor 55 (GPR55), transient receptor potential (TRP) ion channels, and peroxisome proliferator-activated receptors (PPARs). The metabolic enzymes consist of fatty acid amide hydrolase and monoacylglycerol lipase [11,12,13,14]. CB1 and CB2 receptors are the primary mediators of cannabinoid-induced biological effects. They belong to the Gi/o-coupled receptor family, exerting their actions mainly through adenylate cyclase inhibition and potassium channel regulation [11,15].

**Figure 1:**
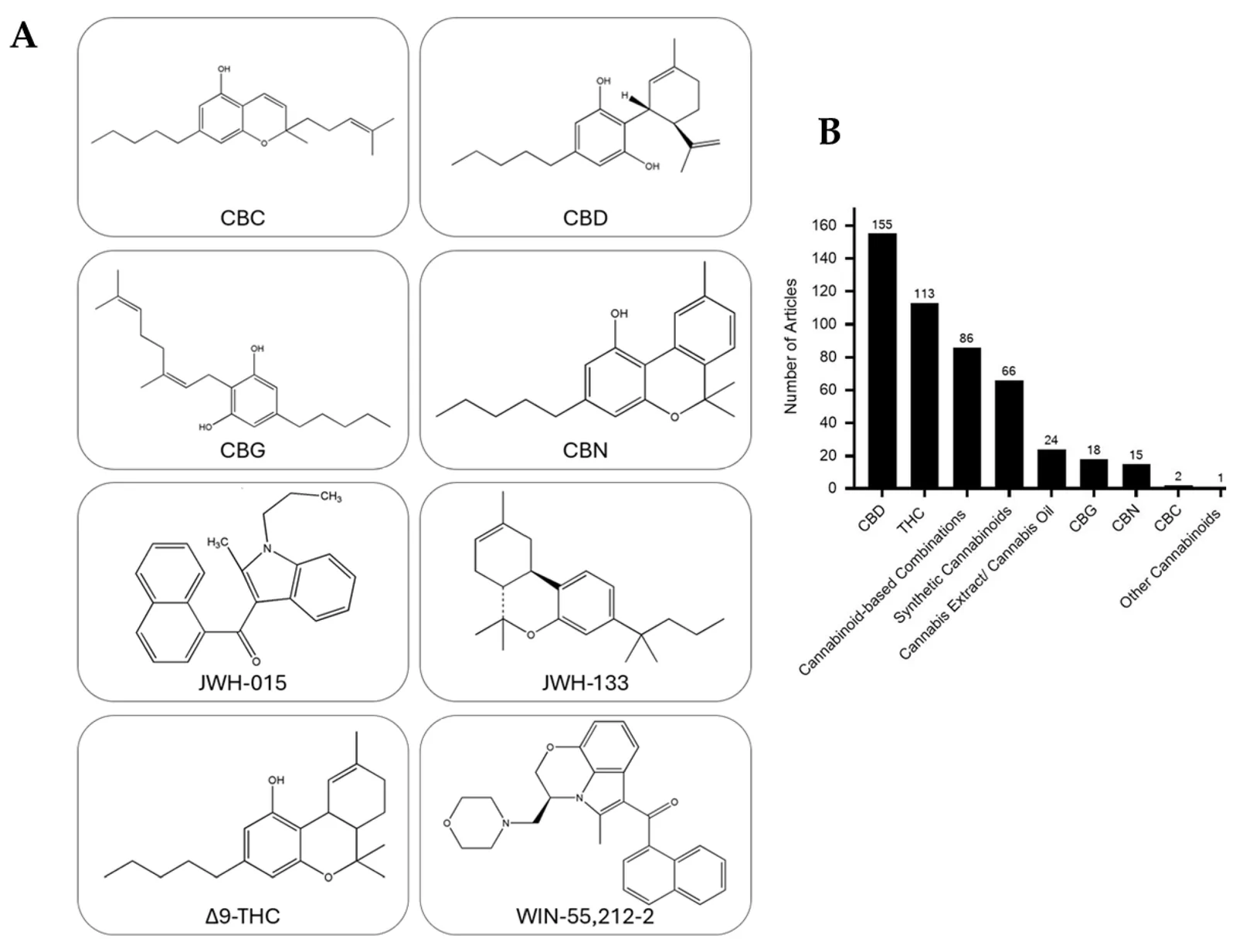
Chemical structure and cancer cell death research landscape of cannabinoids. (**A**) Chemical structures of key phytocannabinoids and synthetic ones. (**B**) A histogram showing the estimated distribution of studies across different cannabinoids, cannabinoid combinations, and cannabis extracts/oils in modulating cancer cell death. The literature search used to generate these estimates included studies published from 1975 through June 2026. THC tetrahydrocannabinol; CBD cannabidiol; CBN cannabinol; CBG cannabigerol; CBC cannabichromene.

Phytocannabinoids constitute another major class of cannabinoids, derived from the cannabis plant. They are classified into different classes based on their chemical structures: Δ^9^ and Δ^8^ -tetrahydrocannabinol (THC), cannabidiol (CBD), cannabinol (CBN), cannabigerol (CBG), cannabichromene (CBC), cannabicyclol, cannabinodiol, cannabielsoin, cannabitriol, and miscellaneous low-abundance cannabinoids [14,16]. Notably, Δ^9^ THC, CBD, CBG, and CBC are considered the major cannabinoids due to their relative abundance in the plant [17]. The chemical structures of these major cannabinoids, along with those of the minor cannabinoid CBN, are shown in Figure 1A. A third class of cannabinoids includes synthetic compounds that share structural similarity to the natural phytocannabinoids, but they generally exhibit enhanced potency, stability, and receptor selectivity, thereby facilitating mechanistic studies of cannabinoids in health and disease and advancing their therapeutic potential [18].

The anticancer activity of cannabinoids was discovered in 1975 by Munson et al. [19]. Cannabinoids suppress cancer cell proliferation, angiogenesis [20], tumor invasion, metastasis, [21], and stemness [22] while inducing different modes of cell death pathways. Cell death is a fundamental biological process that maintains tissue homeostasis and eliminates damaged or abnormal cells. In cancer, the regulation of cell death is often disrupted and represents one of the earliest hallmarks of cancer. Resisting cell death reinforces cancer cell uncontrolled proliferation, survival in adverse microenvironments, and resistance to conventional therapies [23,24]. Therefore, disrupting the mechanisms that enable cancer cells to evade cell death may enhance therapeutic efficacy, promote tumor regression, and improve patient outcomes by facilitating the targeted elimination of malignant cells.

Over the past two decades, research on cannabinoids and their cancer cell death properties has expanded considerably; however, the extent of investigation varies markedly among the different cannabinoid types and formulations (Figure 1B). Our literature search indicates that studies on THC and CBD dominate the field, addressing their effects on cancer cell death with over a hundred publications. In comparison, other phytocannabinoids such as CBG, CBN, and CBC have been explored far less frequently. However, synthetic cannabinoids and cannabinoid-based combinations have been extensively studied for their cancer cell death-modulating effects. Research on whole-plant extracts and cannabis-derived oils remains limited, even though these formulations more closely represent what has been used in clinical settings.

In this review, we first outline the different mechanisms of cell death. We then summarize and evaluate evidence demonstrating how the most extensively studied phytocannabinoids, THC, CBD, CBN, CBG, and CBC, together with selected synthetic cannabinoids, induce cancer cell death across diverse tumor types in both in vitro and in vivo models.

### 1.1. Cell Death Mechanisms

Given the ability of cannabinoids to modulate cancer cell death pathways, a comprehensive understanding of the different cell death modalities is essential to provide a mechanistic framework for explaining and harnessing their anticancer activity. Multiple forms of cell death have been identified, each characterized by specific molecular pathways and morphological features, including apoptosis, autophagy, necrosis, ferroptosis, parthanatos and pyroptosis, among others.

#### 1.1.1. Apoptosis

Apoptosis, also known as type I programmed cell death, is a tightly regulated process in eukaryotic cells characterized by distinct morphological and molecular mechanisms [24]. This mode of cell death is the most characterized and mainly involves cell shrinkage, chromatin condensation (pyknosis), nuclear fragmentation (karyorrhexis), organelle changes, membrane blebbing, and apoptotic body formation, all occurring before plasma membrane integrity is lost [25,26]. Apoptosis is generally non-inflammatory, as cells undergoing apoptosis are rapidly engulfed by phagocytes [27]. This cell death mechanism is energy-dependent, primarily driven by caspase activation, and presents in two pathways [26,28]. The extrinsic pathway is triggered by death ligands (e.g., first apoptosis signal ligand FasL, tumor necrosis factor TNF) binding to receptors (fas cell surface death receptor/ cluster of differentiation 95, Fas/CD95, TNF receptor TNFR1), forming the death inducing signaling complex (DISC) and activating caspase-8/10, which directly stimulate executioner caspases-3/7 [25,26]. However, the intrinsic pathway responds to stress signals namely DNA damage, endoplasmic reticulum (ER) stress, reactive oxygen species (ROS), and mitotic defects inducing mitochondrial outer membrane permeabilization via B-cell lymphoma 2 (BCL-2) family proteins [29]. Pro-apoptotic bcl-2-associated x protein/bcl-2 killer BAX/BAK release cytochrome c and second mitochondria-derived activator of caspases/direct inhibitor of apoptosis protein binding protein SMAC/DIABLO, with cytochrome c, and apoptotic protease activating factor 1 (APAF-1) forming the apoptosome, leading to the activation of caspase-9 [25]. Eventually both apoptotic pathways converge on the executioner caspases 3/7, dismantling cellular components and executing cell death [24,28]. Inhibitors of apoptosis proteins, such as X-linked inhibitor of apoptosis protein XIAP, can inhibit caspases but are neutralized by SMAC [25]. Dysregulated apoptosis can contribute to neurodegenerative diseases, cancer, and autoimmune disorders [30,31].

#### 1.1.2. Necrosis

Necrosis is broadly defined as a form of cell death that was historically considered uncontrolled or unprogrammed, in contrast to apoptosis [32]. Once regarded as an accidental outcome of non-specific stressors, it is now understood that necrosis can occur in a regulated manner, with the Nomenclature Committee on Cell Death recognizing it as a distinct process [25]. Morphologically, necrosis is characterized by swelling and dilatation of cytoplasmic organelles such as mitochondria and the endoplasmic reticulum, along with chromatin condensation into poorly defined clumps, random DNA degradation, and late nuclear alterations such as pyknosis, karyorrhexis, and karyolysis, which is the dissolution of the nucleus resulting in nuclear swelling and loss of affinity for basic dyes [26,28,33]. A defining hallmark of necrosis is early plasma membrane rupture, which results in the release of intracellular contents and evokes a robust inflammatory response, distinguishing necrosis from the immunologically silent nature of apoptosis [24,27]. Necrosis also involves mitochondrial dysfunction, depletion of adenosine triphosphate (ATP), dysregulation of calcium ion (Ca^2+^) homeostasis, and excessive generation of ROS. Proteolytic activity mediated by cathepsins also plays an important role in the dismantling of cellular components [29]. Triggers of necrosis include receptor activation by factors such as TNFR1, Fas, Toll-like receptors, or Z-DNA binding protein 1, as well as diverse cellular stressors including ischemia, trauma, ROS-induced Ca^2+^ overload, lysosomal rupture, iron-driven lipid peroxidation, and DNA damage. Furthermore, inhibition of apoptosis may divert cells toward necrosis, highlighting the interconnectedness of cell death programs [29,31,34]. Importantly, necrosis is subject to regulation by pharmacological and molecular modulators. Caspase inhibition, for example, prevents the execution of apoptosis and can redirect cell fate toward necrosis [31]. Additionally, the cellular antioxidant machinery provides basal protection against ROS-mediated necrotic injury [27]. These findings challenge the early notion of necrosis as merely uncontrolled cell death and instead position it as a context-dependent, modifiable process. Necrosis is prominent in a range of pathophysiological conditions, including ischemia, trauma, neurodegeneration, tumor progression, particularly under hypoxic stress, and chronic inflammatory disorders [26,28,34]. Beyond its detrimental consequences, necrosis can also contribute to shaping the inflammatory microenvironment, influencing tissue remodeling and disease progression [25].

#### 1.1.3. Necroptosis

Necroptosis is a modality of regulated cell death, representing a programmed alternative when apoptosis is inhibited [25,29]. Necroptosis morphologically resembles necrosis in features such as cell swelling, plasma membrane rupture, and inflammation; however, it is driven by molecular pathways. Necroptosis is mediated by receptor-interacting protein kinases 1 and 3 (RIPK1) and (RIPK3), which assemble into the necrosome, leading to phosphorylation of mixed lineage kinase domain-like protein and its translocation to the plasma membrane, thus disrupting membrane integrity and inducing lytic cell death [25]. Necroptosis is increasingly recognized as a major contributor to inflammatory pathologies, bridging the gap between apoptosis and necrosis [25].

#### 1.1.4. Autophagy-Dependent Cell Death

Autophagy-dependent cell death, historically referred to as type II programmed cell death, is a regulated mechanism involving lysosomal degradation and recycling of intracellular components [25,29]. Autophagy-dependent cell death is characterized by extensive autophagic vacuolization and its dependence on core autophagy proteins, namely autophagy-related genes (*Atg*). However, unlike apoptosis, autophagy-dependent cell death is not defined by membrane blebbing and caspase activation. Morphologically, autophagy-dependent cell death displays features including the formation of double membraned autophagosomes that engulf organelles and cytoplasmic material. These autophagosomes then fuse with lysosomes to form autolysosomes for degradation [25]. Mechanistically, autophagy-dependent cell death is initiated in response to cellular stresses such as nutrient deprivation, hypoxia, ER stress, ROS, and drug treatment [31]. The induction of autophagy-dependent cell death begins with the assembly of the UNC-51-like kinase 1 /2–Atg13–Focal adhesion kinase family-interacting protein of 200 kDa (ULK1/2–Atg13–FIP200) complex, tightly regulated by mechanistic target of rapamycin complex 1 mTORC1 (inhibitory) and adenosine monophosphate-activated protein kinase (AMPK) (stimulatory), which phosphorylate ULK1 to control phagophore nucleation [31]. The nucleation step is driven by the Beclin1–VPS34–Atg14–Vps15 complex at ER and other membranes, while Atg9 and vacuole membrane protein 1 mediate lipid transport to the isolation membrane. Autophagosome elongation and closure require two ubiquitin-like conjugation systems: the Atg12–Atg5–Atg16 complex, and the Atg8 (microtubule-associated protein 1A/1B-light chain 3; LC3) lipidation system. LC3 is cleaved by Atg4, activated by Atg7, and transferred to Atg3, before conjugation to phosphatidylethanolamine (PE), generating LC3-II, a hallmark of autophagosome maturation [31]. Fusion with lysosomes is mediated by soluble N-ethylmaleimide-sensitive factor attachment protein receptor proteins, notably syntaxin 17, in cooperation with the homotypic fusion and vacuole protein sorting complex and regulators such as tumor protein 53 (p53)-activated autophagy modulator Pacer [31]. Several enzymes are present within the acidic lysosomal environment, namely hydrolases including cathepsins and acid DNases that degrade proteins, lipids, nucleic acids, and damaged organelles needed to finalize the recycling process [35]. Specific forms of autophagy-dependent cell death include macroautophagy, the sequestration of bulk cytoplasmic components, and mitophagy, the selective elimination of mitochondria [29]. Basal autophagy-dependent cell death supports survival under stress; however, excessive or dysregulated autophagy-dependent cell death shifts the balance toward cell death [25].

#### 1.1.5. Ferroptosis

Ferroptosis is a regulated necrotic process defined by iron-dependent lipid peroxidation and accumulation of lethal ROS [25,36]. Morphologically, cells undergoing ferroptosis display smaller mitochondria with condensed membranes and reduced or absent cristae, while the nucleus remains intact [29]. This mechanism is distinct from apoptosis or necrosis as it does not involve caspase activation or cell swelling but is instead driven by impaired glutathione peroxidase 4 (GPX4) activity and accumulation of peroxidized phospholipids. Ferroptosis plays key roles in degenerative diseases, ischemia reperfusion injury, and cancer [25].

#### 1.1.6. Parthanatos

Parthanatos is a form of regulated cell death that is caused by hyperactivation of poly(adenosine diphosphate ribose) polymerase-1 (PARP-1) in response to extensive DNA damage oxidative stress, and precipitated by bioenergetic catastrophe [25,29]. Unlike apoptosis, parthanatos does not rely on caspases but instead on massive poly adenosine diphosphate-ribose (PAR) production and translocation of apoptosis-inducing factor (AIF) from the mitochondria to the nucleus, where it causes large-scale DNA fragmentation and chromatin condensation [29]. Morphological features of parthanatos include mitochondrial swelling and loss of membrane potential, accompanied by plasma membrane rupture and inflammation. Parthanatos has been implicated in cancer, neurodegenerative diseases, and stroke [25].

## 2. Cannabinoids and Cancer Cell Death

Cannabinoids are promising bioactive compounds that are capable of modulating key signaling pathways that regulate cancer cell survival and death. Their diverse chemical structures and receptor interactions enable them to influence multiple cell death processes, including apoptosis, autophagy, and necrosis, among others.

### 2.1. Tetrahydrocannabinol

THC is the primary psychoactive constituent of the cannabis plants. Evidence from meta-analyses shows that inhaled THC results in dose-dependent psychoactive effects, elevating individuals’ reported feelings of intoxication “feeling high”, calmness, tiredness, and anxiety while decreasing alertness [37]. THC exists in several structurally related forms, the most common being the positional isomers Δ^9^-THC and Δ^8^-THC. These isomers chemically differ by the position of a double bond at C8-C9 in Δ^8^-THC and C9-C10 in Δ^9^-THC. Δ^8^-THC which is primarily isolated from dried cannabis leaves and flowers in the United States of America (USA), is more stable but less abundant in the plant [38,39]. In contrast, Δ^9^ -THC is one of the major constituents of *Cannabis sativa* resin produced by the leaves and buds of the female plant flowers [14]. This latter isomer has demonstrated significant suppression of cancer cell viability and tumor growth across various cancer types [40]. The mechanisms of cell death induced by THC in various cancer types are summarized in Table 1. The main receptors that Δ^9^-THC interacts with are the CB1 and CB2, GPR55, TRPVs, and to some extent PPARγ [41]. Δ^9^-THC mediates apoptosis in endometrial cancer cells through upregulating the BCL-2 family member Noxa, inducing ROS accumulation and downregulating *RAF1* gene expression [42]. THC, in both of its forms -Δ^9^ and Δ^8^-, decreases oral cancer cell viability at IC_50_ 10 μg/mL and 13 μg/mL, respectively, along with inducing apoptosis-dependent mitochondrial membrane depolarization, and increases both caspase 3 and caspase 9 activity and expression, BAX/BCL-2 ratio as well as autophagy-dependent cell death [43]. In human colorectal cancer (CRC) cell lines, treatment with Δ^9^-THC at approximately 50 μM concentrations for SW480 cells and LoVo cells leads to increased expression of cell death markers, including p53, cleaved PARP-1, RIP1, and RIP3, along with the upregulation of BAX and downregulation of BCL-2 [44]. Furthermore, 10 μM of THC induces ceramide accumulation, mitochondrial dysfunction, cytochrome c release, and caspase activation, ultimately resulting in apoptosis in Jurkat acute T cell leukemic cells [45]. In melanoma cells, THC treatment induces apoptosis marked by a downregulation of aryl hydrocarbon receptor nuclear translocator 2 (*ARNT2*), cyclin E2 (*CCNE2*), integrin subunit alpha 9 (*ITGA9*), proliferating cell nuclear antigen (*PCNA*), and E2F transcription factor 1 (*E2F1*) genes. On the other hand, THC treatment of melanoma cells results in the upregulation of DNA damage inducible transcript 3 (*DDIT*), nerve growth factor receptor (*NGFR*), colony-stimulating factor 2 (*CSF2*), growth arrest and DNA damage inducible beta (*GADD45B*), and thymic stromal lymphopoietin genes [46].

The stress-regulated protein 8 (p8), also known as the nuclear protein 1, is primarily localized in the nucleus and functions as a stress-inducible transcription factor. Cellular stressors inducing p8 functions include ER stress, hypoxia, glucose deprivation, DNA damage, and oxidative stress [47]. In the context of cancer, based on the cell type and stress status, p8 can play a dual role: either as an oncoprotein promoting tumor survival and drug resistance or as a tumor suppressor by mediating ER stress-induced apoptosis [48,49]. For example, p8 can function through the protein kinase R-like ER kinase-eukaryotic initiation factor 2 alpha-activating transcription factor 4-p8-C/EBP homologous protein (PERK–eIF2α–ATF4–CHOP) axis ultimately leading to mitochondrial-mediated cell death. p8 is a key mediator of THC-induced apoptosis. THC treatment in multiple cancer cell types, such as astrocytoma U87MG, oligodendroglioma GOS3, pancreatic cancer MiaPaCa2, and melanoma A375, decreases cell viability, activates caspase 3, and upregulates p8 expression [50]. Notably, tumors deficient in p8 exhibit resistance to THC’s pro-apoptotic effects, demonstrating its critical role in mediating THC’s anticancer activity. Mechanistically, THC binding to the cannabinoid receptors triggers de novo ceramide synthesis, activation of ER stress-related genes such as *inducing transcription factor 4*, *C/EBP homologous protein*, and *tribbles pseudokinase 3*, ultimately driving apoptosis [50]. Similarly, cannabinoid receptors activation by Δ^9^-THC leads to the activation of rapidly accelerated fibrosarcoma 1/extracellular signal-regulated kinase (Raf1/ERK) signaling, and subsequent generation of ROS [51]. The accumulation of ROS induces genotoxic stress, which triggers apoptosis in glioma cells both in vitro and in vivo [52]. Ceramide-mediated ER stress also enhances the expression of p8, further activating caspase cascades to promote apoptosis and autophagy [53].

In transformed mouse embryonic fibroblasts, a THC treatment at 6 μM results in the complete loss of cell viability and increases the expression of autophagic markers, which precedes those of apoptotic features [54]. Interestingly, inhibition of autophagy-dependent cell death prevented THC-induced apoptosis, demonstrating that autophagy-dependent cell death can act as an upstream regulator of apoptosis [55]. In melanoma cells, THC-induced autophagy-dependent cell death was independent of beclin-1, suggesting a noncanonical autophagy-dependent cell death mechanism [55]. Noncanonical autophagy-dependent cell death pathways appear to interact with the apoptosis machinery, promoting both caspase-independent cell death and apoptosis [56]. Ultimately, these studies reveal that THC exerts its anticancer effects through multiple mechanisms, including apoptosis, autophagy, and oxidative stress, mediated by cannabinoid receptor activation, ceramide accumulation, and ER stress pathways. The synergistic potential of THC with other cannabinoids and chemotherapeutic agents further emphasizes its promise as a multifaceted anticancer agent with broad applicability across diverse cancer types [40].

Although THC is known to reduce cancer cell viability and is associated with apoptotic markers characterized by increased cytochrome c release, BAX/BCL2 ratio, and caspase activation, this model is not universal. Some findings show that THC’s antitumor effects are associated with ceramide accumulation and ER stress, while other studies demonstrate that they are associated with mitochondrial dysfunction and elevated ROS levels, highlighting the diversity of responses triggered by THC that may lead to cell death. In addition, both apoptosis and autophagy-dependent cell death could be triggered by THC. Importantly, the variable outcomes observed across cancer models may reflect differences in tumor lineage and genetic background, which can influence ER stress responses, basal ROS levels, mitochondrial homeostasis, and the expression of key regulators, including p8 and BCL-2 family proteins. Experimental variables, particularly THC concentration, treatment duration, and model-specific conditions, may further contribute to these differential responses. Despite accumulating preclinical evidence, in vivo studies remain limited, precluding definitive conclusions regarding the predominant cell death mechanism induced by THC. Additional studies across well-characterized animal models are therefore needed to clarify the relative contribution of individual cell death pathways to the antitumor effects of THC.

**Table 1:** Roles and mechanisms of cannabinoids in promoting cell death in various cancer types and models. AIF apoptosis-inducing factor; AKT protein kinase B; ARNT2 aryl hydrocarbon receptor nuclear translocator-like protein 2; ATF4 activating transcription factor 4; *ATG* autophagy-related genes, ATG5 autophagy-related protein 5; ATG16 autophagy-related protein 16; Bax Bcl2-associated X protein; Bcl-2 B-cell lymphoma 2; BiP binding immunoglobulin protein; CBC cannabichromene; CBG cannabigerol; CBD cannabidiol; CBN cannabinol, CCNE2 cyclin E2, CDK2 cyclin-dependent kinase 2; CHOP C/EBP homologous protein; CSF2 colony-stimulating factor 2; DDIT DNA damage-inducible transcript; DUSP1 dual specificity phosphatase 1; E2F1 E2F transcription factor 1; EGFR epidermal growth factor receptor; eIF2α eukaryotic initiation factor 2 alpha; ER endoplasmic reticulum; ERK extracellular signal-regulated kinase; FAK focal adhesion kinase; GPX4 glutathione peroxidase 4; GSH glutathione; GSC glioblastoma stem-like cell; H2AX histone H2A variant X; HCC hepatocellular carcinoma; HNSCC head and neck squamous cell carcinoma; HSP70, heat shock protein 70; ITGA9 integrin subunit alpha 9; JNK c-Jun N-terminal kinase; Keap/Nrf2 Kelch-like ECH-associated protein/nuclear factor erythroid 2-related factor 2; LC3 microtubule-associated protein 1 light chain 3; LC3-II LC3-phosphatidylethanolamine conjugate; MAPK mitogen-activated protein kinase; MEF mouse embryonic fibroblast; mTOR mechanistic target of rapamycin; mTORC1 mechanistic target of rapamycin complex 1; NFκB nuclear factor Kappa light-chain-enhancer of activated B cells; NGFR nerve growth factor receptor; NSG NOD scid gamma; p53 tumor protein p53; p8 nuclear protein 1 NUPR1; p38 MAPKs p38 mitogen-activated protein kinases; PARP-1 poly ADP-ribose polymerase-1; PARP-1 poly(ADP-ribose) polymerase 1; PCNS proliferating cell nuclear scaffold; PPARγ peroxisome proliferator-activated receptor gamma; Rac1/Cdc42 Rac family small GTPase 1/cell division cycle 42; Ras rat sarcoma viral oncogene homolog; RIP1 receptor-interacting protein kinase 1; RIP3 receptor-interacting protein kinase 3; ROS reactive oxygen species; SLC7A11 solute carrier family 7 member A11; Smac second mitochondria-derived activator of caspases; TFRC transferrin receptor protein 1; THC Δ9-tetrahydrocannabinol; TRIB3 tribbles pseudokinase 3; TRPV1 transient receptor potential vanilloid 1; TRPV4 transient receptor potential vanilloid 4; *Tsg101* tumor susceptibility gene 101; XIAP X-linked inhibitor of apoptosis protein.↑, increase; ↓, decrease; →, leads to.

Cannabinoid	Cancer Types	Cancer Models	Cell Death	Mechanism	Reference
THC	Oncogene-transformed mouse embryonic fibroblasts	Transformed MEF from TRIB3^-/-^ mice	Autophagy Apoptosis	↑ ER stress-related proteins (TRIB3) ↓ AKT/MTORC1 ↑ in LC3-II expression ↑ caspase-3 expression	[54]
	Colorectal cancer	SW480 and LoVo cells	Apoptosis	↑ p53 ↑ PARP-1 ↑ RIP-1, RIP-3 ↑ Bax/Bcl2	[44]
	Leukemia	Jurkat cells	Apoptosis	↑ cytochrome c ↑ caspase 3 activation	[45]
	Melanoma	A375 cells	Apoptosis	↑ DDIT, NGFR, CSF2 ↓ ARNT2, CCNE 2, ITGA9, PCNA	[46]
	Other types	Astrocytoma U87MG Oligodendroglioma GO33 Pancreatic cancer MiaPaCa2 Melanoma A375	Apoptosis	↑ activated caspase 3 ↑ p8 expression	[50]
THC + CBD	Breast cancer	MDA-MB-231 and MCF-7 cells	Apoptosis	↑ cytochrome c ↑ Bax/Bcl2	[57]
THC + CBC	Bladder cancer	T24 and HTB-9 cells	Apoptosis	↑ ceramide ↑ caspase 3 activation	[58]
THC/CBD + Temozolomide	Glioblastoma	Glioblastoma xenografts	Autophagy Apoptosis	↑ ceramide synthesis by THC ↑ ROS by CBD	[59]
THC/CBD (Sativex^®^)	Melanoma	CHL-1, A375, SK-MEL-28 melanoma cells	Autophagy Apoptosis	THC-induced apoptosis requires TRIB3 and is mediated by ATG7-dependent autophagy	[55]
THC + JWH-015	Hepatocellular carcinoma	Mice xenografts-induced HCC tumors	Apoptosis Autophagy	↑ upregulation of PPAR-γ-dependent pathways	[60]
CBD	Breast Cancer	MCF-7 cells	Apoptosis	↑ Bcl-2/caspase-3 pathway (CBD and F1 specific) ↓ FAK, Akt, ERK1/2, p38MAPKs, NF-κB (upstream), and Rac1/Cdc42 (downstream) ↓ mTOR	[61]
		MCF-7 and MDA-MB-231 cells Non-tumorigenic MCF10A cells	Apoptosis	↑ calcium through TRPV1 ↑ ER stress ↑ ROS production	[62]
		MDA-MB-231 and MDA-MB-468 cells	Autophagy	↓ fibronectin, vimentin, and integrins (-alpha5, -beta5, -beta1) ↓ beclin-1 and ATG5, 7, 16.	[63]
	Lung cancer	A549 cells	Apoptosis	↑ apoptosis-related proteins (p53, PARP, RIP1, RIP3, ATG12, Beclin). ↑ intracellular vesicle formation regulated by PPARγ. ↑ E-cadherin and vesicle-formation components (clathrin, β-adaptin, Tsg101).	[64]
	Colorectal cancer	HT-29, SW480, HCT-116, and HCT-15 cells	Apoptosis Paraptosis Autophagy	↑ MAPK pathway (JNK, p38, ERK) ↓ anti-apoptotic proteins and ↑ annexin-V-positive and TUNEL-positive cells ↑ ATF4 and CHOP expression, ER stress, and ROS generation ↑ Atg7, phospho-Beclin-1, and LC3	[65]
		HCT116 p53wt, LS174T p53wt, SW480 p53mut, and HCT116 p53 (-/-) cells	Apoptosis	↑ ROS production (p53wt cells only) ↑ ROS ↑ keap1/Nrf2 pathway ↑ p53-dependent caspase-8/9/3 activation ↑ PARP1, ↑ G0/G1 cell cycle arrest; ↓ CDK2. ↓ Hsp70	[66]
		HT-29 cells	Apoptosis Necrosis	↑ oxidative stress via CB receptor-independent mechanisms	[67]
	Ovarian cancer	A2780 cells	Apoptosis	loss of mitochondrial membrane potential, Annexin V, caspase 3/7, ROS activities	[68]
	Glioma and Glioblastoma	U87 and U373 cells	Autophagy Ferroptosis	↑ ERK signaling (not JNK and p38), ↑ ROS production ↑ LC3 II, Atg7, and beclin-1 ↑ p-ERK → ↓ GPX4 ↓ SLC7A11 ↑ TFRC ↑ ER stress, iron load, ↓ GSH levels.	[69]
		Human glioma cells: U87 MG, LN18, U118 MG Patient-derived glioma sphere culture Mouse orthotopic models	Autophagy Mitophagy	mitochondrial dysfunction and lethal mitophagy arrest ↑ calcium flux through TRPV4 ↑ ER stress and the ATF4-DDIT3-TRIB3-AKT-MTOR axis downstream of TRPV4 Combination with temozolomide shows synergistic effects in patient-derived neurosphere cultures and mouse orthotopic models.	[70]
		Glioblastoma stem-like cells GSC lines (#1, #30 and #83) previously characterized isolated from surgical samples of three adult patients.	Autophagy Apoptosis	↑ TRPV2 activation ↑ Aml-1a	[71]
	Gastric cancer	SGC-7901 cells	Apoptosis	↑ ataxia telangiectasia mutated gene and p53 protein. ↓ CDK2 and cyclin E ↑ ROS levels ↑ Bax expression ↓ Bcl-2 levels ↓ mitochondrial membrane potential, ↑ caspase-3 and caspase-9	[72]
		Gastric carcinoma cells: AGS, MKN45, MKN74, SNU638, NCI-N87 Normal gastric cells: HFE-145 Xenograft mouse model injected with MKN45 gastric cancer cells	Apoptosis	↑ ER stress. ↑ Smac release from mitochondria ↑ ubiquitination XIAP → ↓ XIAP	[73]
	Endometrial cancer	Ishikawa, MFE-280 (well and poorly differentiated type I) HEC-1A (moderately differentiated type I) PCEM002 (poorly differentiated type I) PCEM004a and PCEM004b (poorly differentiated mixed type I/II cell lines).	Apoptosis Autophagy	↑ p-Akt ↑ Annexin V	[74]
	Cervical cancer	HeLa, a metastatic ME-180, and a primary SiHa cell line	Apoptosis	↑ p53, caspase 3, and Bax	[75]
	Prostate cancer	PC3 cells	Apoptosis	↑ caspase 3/7 ↑ DNA fragmentation ↑ Bax expression, ROS production, and altered mitochondrial membrane potential. ↓ glutathione and ATP levels. ↑ oxidative stress	[76]
	Other types of cancer	KKU-213B and gemcitabine-resistant cholangiocarcinoma cells Gemcitabine-resistant cholangiocarcinoma xenograft model	Apoptosis	↑ ROS production. ↑ Expression of ER stress-associated apoptosis proteins, including p-PERK, BiP, ATF4, CHOP, BAX, and cytochrome c ↑ CHOP and ↓ PCNA in xenografted mouse	[77]
		OEC-M1 oral squamous cell carcinoma cells	Apoptosis	-	[78]
		FaDu (KCLB-30043), Hep2 (KCLB-10023), SNU-899 (KCLB-00899) and SCC15 human HNSCC cells In vivo: Xenograft mouse models	Autophagy Apoptosis	↑ apoptosis and autophagy potentially mediated by the activity of DUSP1. ↓ migration/invasion and cell viability ↑ chemotherapy drug efficacy	[79]
CBD + CBG	Glioblastoma	U87, U373, and T98 cells	Apoptosis	Caspase 3 activation	[80]
CBD + CBG + CBN	Ovarian cancer	HTB75 and HTB161 cells	Apoptosis	↑ cleaved caspase 3 ↑ cleaved PARP ↑ DNA fragment	[81]
CBN	Lung cancer	A549 cells	Apoptosis	↓ EGFR tyrosine kinase → ↑ Anticancer effect ↑ Apoptosis	[82]
	Myeloma	U266B1, RPMI-8226, and SKO-007 cells	Necrosis	↑ DNA damage (↑ protein level of γ-H2AX) ↑ Necrotic cell death (↑ % propidium iodide positive cells)	[83]
		Xenograft mouse model: B-NDG mice subcutaneously injected with U266 multiple myeloma cells		Reducing tumor weight	
	Neuroblastoma	SHSY-5Y cells	Apoptosis	↓ Cell viability, ↑ activation of caspases 3/7 ↑ Apoptosis	[15]
		IMR-5 and SK-N-AS cells	Apoptosis	↓ AKT pathway ↑ miR-34a → → ↓ E2F1 → ↓ cell cycle	[84]
	Cholangiocarcinoma	HuCCT1 cells	Apoptosis	↓ AKT, MAPK pathways ↓ AKT, GSK-3α/β, and ERK 1/2 phosphorylation ↑ PARP cleavage, ↑ Annexin positivity	[85]
CBG	Lung cancer	A549 lung adenocarcinoma cells	Apoptosis	↓ EGFR tyrosine kinase	[86]
	Epidermoid carcinoma	A431 epidermoid carcinoma cells			
	Ovarian cancer	A2780 (chemotherapy-sensitive) and A2780/CP70 (chemotherapy-resistant) human ovarian carcinoma cells	Apoptosis	↑ caspase 3/7 activation	[68]
	Colorectal cancer	Caco-2 and HCT116 cells	Apoptosis	↑ reactive oxygen species ↑ oxidative stress	[87]
	Pancreatic cancer	PANC-1 and MIAPaCa-2 pancreatic ductal adenocarcinoma cells	Autophagy Apoptosis	↓ EGFR ↓Akt/mTOR ↓ Ras ↑ LC3-I conversion to LC3-II, ↑ caspase-3/procaspase-3 ↑ Annexin V^+^ cells	[88]
	Cholangiocarcinoma	HuCC-T1 cells	Apoptosis	↑ Bax/Bcl-2 ratio ↑ cleaved caspase-3	[89]
	Glioma	U87, NCH644, NIB138, K26, and T98 cells A-172 cells	Apoptosis	↑ activated caspase-3 ↑ p-ERK1/2, ↓ *p*-AKT 1/2/3	[86]
	Myeloma cells	U266B1, RPMI-8226, and SKO-007 cells	Necrosis	↑ DNA damage (↑ γ-H2AX) ↑ % propidium iodide positive cells	[83]
Synthetic Cannabinoids					
WIN-55	Acute myeloid leukemia	HL60, MV-4–11 and KG-1a cells Immunocompromised NSG mice injected intravenously with HL60 cells	Apoptosis Parthanatos	↑ caspase 3 cleavage ↑ PARP cleavage ↓ procaspase -2, -8, and -9 ↑ p-JNK, ↑ p-Erk1/2, ↓ p-p38-MAPK, ↓ p-AKT ↑ long (C16: 0-C20: 1) and very long chain ceramides (C22: 0-C24: 1) ↓ protein levels of AIF in the mitochondria	[90]
WIN-55	Prostate cancer	LNCaP cells	Apoptosis	↑ Bax/Bcl-2 ratio, ↑ PAPR cleavage	[91]
	Glioma	Rat C6 cells	Apoptosis	↓ phosphorylated Bad levels ↑ mitochondrial depolarization ↑ activation of caspase cascade ↓ AKT and Erk pathways	[92]
JWH-133	Melanoma	B16 melanoma xenografted mice	Apoptosis	-	[93]

### 2.2. Cannabidiol

CBD is a major phytochemical found in the *Cannabis sativa* plant. This phytocannabinoid attracted scientific interest due to its pharmacological and medical non-psychoactive properties [94]. CBD is generated by the decarboxylation of cannabidiolic acid during heating, smoking, vaporization, or baking of dried cannabis flowers [95]. Initially, CBD’s primary medical application was to manage seizures, particularly in severe and treatment-resistant epilepsy [96]. In 2018, the Food and Drug Administration (FDA USA) approved Epidiolex, a CBD-based oral solution, for treating Lennox–Gastaut and Dravet syndromes [97]. Recent studies indicate that CBD exhibits neuroprotective, anti-inflammatory, and anxiolytic effects, as well as anticancer mechanisms that are dependent on cancer type [98,99]. These mechanisms operate through both receptor-dependent and -independent pathways across various cancer cell lines [99]. The cell death mechanisms induced by CBD in various cancer types are detailed in Table 1.

CBD has low affinity for CB1 and CB2 receptors but interacts with other receptors like GPR55, TRP vanilloid type 1 (TRVP1), and PPARs influencing various cancer-related signaling pathways, which are involved in increased cell proliferation and tumor progression [100,101]. These effects result in the inhibition of tumor cell growth, induction of cell death, and suppression of tumor angiogenesis [79]. CBD’s potent antitumor activities in breast cancer are highlighted by Almeida et al. [102]. Recent studies have demonstrated that CBD suppresses the migration and invasion in the estrogen receptor-positive (ER+) MCF7 breast cancer cells by modulating the FAK/MAPK/AKT/NF-κB signaling pathways and induces apoptosis via the BCL-2/caspase-3 axis [103]. Furthermore, CBD induces oxidative stress and activates the unfolded protein response in MCF7 cells but not in the triple-negative breast cancer (TNBC) MDA-MB-231 cells. This increased oxidative stress in MCF7 cells was attributed to calcium influx through the activation of the TRPV1 receptor [62]. Notably, CBD has been observed to induce programmed cell death in breast cancer cells by coordinating the crosstalk between apoptosis and autophagy-dependent cell death independently of cannabinoid and TRVP1 receptor pathways. Beclin1 links apoptosis and autophagy-dependent cell death and plays a central role in coordinating their crosstalk [61]. Free beclin-1 may initiate autophagy-dependent cell death through its interaction with the class III PI3K complex (Vps34); however, its binding to the anti-apoptotic protein bcl-2 suppresses autophagy-dependent cell death. CBD improves the association between beclin-1 and Vps34 while disrupting the interaction between beclin-1 and bcl-2, thus mediating autophagy-dependent cell death [104]. In parallel, CBD activates intrinsic apoptosis in breast cancer cells by dissipating the mitochondrial membrane potential, triggering BID mitochondrial translocation, and promoting cytochrome c release into the cytosol. Finally, CBD elevates ROS levels which are implicated in the induction of both apoptosis and autophagy-dependent cell death [61]. While CBD typically promotes apoptosis and autophagy-dependent cell death [105], a study indicated that CBD inhibits autophagy-dependent cell death in MDA-MB-231 and MDA-MB-468 TNBC cells by reducing beclin-1 and Atg-5, Atg-7, and Atg-16 levels. It also showed that CBD reduced migration and invasion and enhanced doxorubicin sensitivity [63], highlighting the double-edge sword of autophagy-dependent cell death in cancer progression [106]. Collectively, these studies suggest that autophagy-dependent cell death may initially function as a cytoprotective response to CBD-induced cellular stress, under sustained or excessive stress, may become dysregulated, and may contribute to autophagy-dependent cell death. Importantly, changes in the expression or accumulation of autophagy-related markers alone cannot be considered sufficient evidence that CBD-induced cell death is autophagy-dependent. Establishing the contribution of autophagy-dependent cell death in cancer cells, therefore, requires mechanistic and functional approaches, including assessment of autophagic flux and demonstration that genetic or pharmacological inhibition of the autophagic machinery attenuates CBD-induced cell death.

Another recent study showed that CBD selectively suppresses the viability of human epidermal growth factor receptor 2 (HER2)-positive breast cancer cells and mediates HER2-dependent cytotoxicity through the activation of non-apoptotic cell death pathways. Specifically, CBD triggered both paraptosis, evidenced by reduced ALG-2-interacting protein X and increased ATF4 and CHOP expression, and ferroptosis, characterized by the downregulation of GPX4 and the solute carrier family 7 member 11 and upregulation of transferrin receptor 1 (TFRC). Molecular docking and molecular dynamics analyses revealed a potential interaction between CBD and HER2, elucidating further mechanistic evidence into CBD’s antitumor effect in HER2-positive breast cancer cells [107]. CBD elevates the Bax/Bcl-2 ratio, promoting the release of pro-apoptotic proteins from the mitochondria, and induces ROS production in ovarian cancer cells [68]. CBD has also shown efficacy in suppressing endometrial cancer cell growth by inducing apoptosis, enhancing autophagy-dependent cell death, and increasing sensitivity to chemotherapy, particularly in TRPV2-overexpressing cells, which are associated with poor prognosis [74]. This phytocannabinoid also effectively induced apoptosis in cervical cancer cells, as evidenced by increased expression of p53, caspase-3, and BAX [75]. Furthermore, CBD suppressed the proliferation and invasion of M14 and A375 melanoma cells while significantly promoting apoptosis. Mechanistically, these effects were mediated through the formation of a PPARγ-ten-eleven translocation 1 complex, which induced the demethylation and subsequent upregulation of *leucine-rich repeat* and *sterile alpha motif-containing 1*, a tumor suppressor gene. The increased expression of this gene in turn induced apoptosis and inhibited melanoma cell proliferation and progression [108]. Bioinformatic docking analyses indicated that CBD may interact with epidermal growth factor receptor (EGFR), RAS/RAF isoforms, and MEK1/2 and ERK1/2. These interactions may suppress kinase activities by interfering with phosphoryl transfer required for substrate activation or by binding to the conserved DFG motif within the activation loop of MAPK kinases, thereby reducing their catalytic activity [65]. However, in HT-29, SW480, HCT116, and HCT-15 CRC cells, CBD can also activate the MAPK pathway leading to the induction of apoptosis, paraptosis, and autophagy-dependent cell death through the activation of JNK, p38, and ERK pathways [65], thereby highlighting the heterogeneity of CBD’s mechanisms of action in CRC cells. Furthermore, a study by Wang et al. using wild type, knockout, and mutant p53 human CRC cells, provided new insights into the mechanisms of CBD-induced mutual antagonism of apoptosis and macroautophagy [66]. This study revealed that CBD triggers ROS production in wild-type p53 cells, leading to p53-dependent apoptosis and macroautophagy via pathways involving PARP1 and Kelch-like ECH-associated protein 1-nuclear factor erythroid 2-related factor 2 (keap1/Nrf2). In addition, CBD’s inhibition of heat-shock protein 70 enhances apoptosis and suppresses autophagy-dependent cell death [66]. This study demonstrates that p53 status influences the contribution of ROS-associated stress to the induction of apoptosis and macroautophagy, as well as the functional interplay between these processes, thereby contributing to differential responses among CRC cancer models. Taken together, these findings underscore p53 status as an important genetic determinant of cannabinoid-induced cell death and highlight the broader potential influence of tumor genetic context on cannabinoid responsiveness. HT-29 cells treated with CBD are subjected to redox imbalance and necrosis [67]. Necrosis was confirmed by a significant increase in propidium iodide (PI) positivity in annexin V/PI assays and by transmission electron microscopy showing altered chromatin structure, cytoplasmic changes, plasma membrane disruption, and highly damaged organelles.

In A549 human lung cancer cells, CBD inhibits growth at concentrations above 20 μM by increasing apoptosis-related proteins and promoting intracellular vesicle formation regulated by PPARγ [64]. Inhibition of PPARγ reduces the antiproliferative effects of CBD. This phytocannabinoid has also been shown to exert antitumor activity in drug-resistant cancers, including gemcitabine-resistant cholangiocarcinoma, by inducing stress-associated apoptosis both in vitro and in vivo [77].

Contrary to the previously assumed CB1 and CB2 receptor-dependent mechanisms, Torres and colleagues identified the TRPV2-dependent antiproliferative and pro-apoptotic effects of CBD on glioma [59], consistent with findings from another study [71]. Additionally, CBD was shown to induce ERK activation and ROS production to promote autophagy-dependent cell death and ferroptosis in glioblastoma cells [69]. Mechanistically, CBD elevated the protein expression of several autophagy-dependent cell death players including microtubule-associated protein 1A/1B-light chain 3, form II (LC3 II), Atg7, and beclin-1 in a concentration-dependent manner leading to autophagy-dependent cell death. Also, CBD reduced the expression of the antiferroptosis enzyme GPX4 and that of SLC7A11, the light chain subunit of the cystine/glutamate antiporter system Xc^−^, further disrupting GPX4 activity. Concomitantly, CBD increased the expression of TFRC, thereby promoting lipid peroxidation and ultimately sensitizing glioblastoma cells to ferroptosis [69]. CBD also reduced the mitochondrial membrane potential from approximately 50 to 23% in both U87 MG and U373 cells.

Another study using U87 MG, LN18, U118 MG, A172, and U251 glioma cell lines demonstrated that CBD induces autophagy-dependent cell death through mitochondrial dysfunction and lethal mitophagy arrest rather than apoptosis [70]. This process is initiated by Ca^2+^ flux via the TRPV4 receptor, which triggers mitophagy. Additionally, CBD-mediated mitophagy is regulated by the ATF4-DDIT3- tribbles homolog 3TRIB3-AKT-MTOR signaling pathway downstream of TRPV4 [70]. Recent studies have shown that CBD induces several cell death mechanisms in various cancer models, including apoptosis in prostate cancer [76,109], gastric cancer [72,73], and oral squamous cell carcinoma cells [78,110]. Other studies have documented that CBD induces both apoptosis and autophagy-dependent cell death in head and neck squamous cell carcinoma cells [79].

CBD exhibits low toxicity [62,73] and favorable suitability for combination therapies, making it a promising candidate for sustainable cancer treatment strategies, as co-administration with other agents has been shown to enhance therapeutic efficacy [63,70]. 

The expression levels and distribution of cannabinoid-responsive receptors represent important determinants of the signaling pathways engaged by CBD and may contribute to variability in cellular responses. Given its relatively low affinity for the canonical cannabinoid receptors CB1 and CB2, CBD can modulate several alternative molecular targets, including TRPV1, TRPV2, TRPV4, and PPARγ. The involvement of TRPV1 in breast cancer, TRPV2 in glioma and endometrial cancer, and TRPV4 in glioma-associated mitophagy illustrates how differences in target expression across tumor types may favor distinct signaling networks and cell death mechanisms. Thus, tumor-specific receptor and molecular target profiles may partly account for the heterogeneous anticancer responses to CBD observed across different experimental models.

### 2.3. Cannabinol

CBN is a minor phytocannabinoid primarily produced from the aromatization of the methyl moiety of Δ^9^-THC through oxidative degradation upon exposure to light, heat, or oxygen [17]. CBN is mostly unique for its high stability and mild psychoactive properties. It acts as an agonist for CB1 and CB2 receptors with varying affinities and can also interact with TRP channels [11,17]. Previous evidence has reported several pharmacological effects of CBN including its anti-inflammatory, antibacterial, and orexigenic activities, and it is widely used for pain relief [111,112,113,114] (Table 1). CBN reduced the viability of U266B1, RPMI-8226, and SKO-007 multiple myeloma cells, caused DNA damage, and induced necrosis while reducing tumor weight in U266B1 multiple myeloma xenografted mice [83]. CBN, but not its acetylated derivative that is synthesized to potentially enhance potency, selectivity, and/or efficacy, reduced SHSY-5Y neuroblastoma cell viability and activated caspases 3/7, thereby triggering apoptosis [15]. Consistently, CBN decreased IMR-5 and SK-N-AS neuroblastoma cell proliferation in a dose-dependent manner, reaching a “killing effect” at high doses making its effects on cell death dominate over cell proliferation [84]. Also, CBN inhibited the AKT pathway and upregulated miR-34a, a microRNA that targets E2F1, arresting the cell cycle and stimulating apoptosis [84]. CBN may upregulate phosphorylated ERK1/2 (p-ERK 1/2) levels in A-172 human glioma cells and HepG2 hepatocellular carcinoma cells while it downregulated these levels in HCC1806 breast cancer cells with a decrease in the p-AKT1/2/3 levels in the three cell lines. These latter effects, together with CBN modulation of CB receptors, cyclin-dependent kinases, and cyclins levels, result in cancer cell cycle arrest and apoptosis in a cell- and dose-dependent manner [86]. In addition, CBN induced apoptosis in HuCCT1 human cholangiocarcinoma cells by suppressing the AKT and MAPK pathways, glycogen synthase kinase-3 α and β, and ERK1/2 phosphorylation levels [85]. Since EGFR is overexpressed in multiple cancer types, a study suggested that CBN binds and inhibits EGFR tyrosine kinases, leading to anticancer activity and apoptosis in A549 human lung adenocarcinoma and A431 human epidermoid carcinoma cell lines [82].

### 2.4. Cannabigerol

CBG is a non-psychoactive compound first isolated from *Cannabis sativa* in 1964 and is a byproduct of the non-enzymatic decarboxylation of cannabigerolic acid. Its monocyclic structure, comprising a core dihydropyran and a phenol ring, was proposed by Gaoni and Mechoulam in 1971 [115]. CBG can interact with the CB receptors, exhibiting limited direct activation of CB1 and inhibiting AEA uptake, while acting as a partial agonist at the CB2 receptor [116]. Additionally, it interacts with TRP channels and PPARs [117,118]. Unlike some cannabinoids, CBG acts as a potent α2-adrenoceptor agonist and a moderately potent 5-hydroxytryptamine subtype 1A receptor antagonist, where the latter interaction accounts for CBG’s distinguished neuroprotective effects [119]. CBG also displays anti-inflammation, hypotension, and analgesic roles among others [120].

Compelling evidence confirmed that CBG has potent anticancer activities in diverse cancer types as summarized in Table 1. Upon CBG treatment, PANC-1 and MIAPaCa-2 pancreatic ductal adenocarcinoma cells undergo both autophagy-dependent cell death and apoptosis [118]. These tumor cells exhibited a significant increase in the autophagy-dependent cell death marker LC3-II, which is the lipidated form of LC3-I, suggesting the activation of early autophagy-dependent cell death steps, with an increase in the acidic vesicular organelles that are indicative of full blast autophagy-dependent cell death. The latter antitumor effects of CBG are mediated through the inhibition of the EGFR, AKT/mTOR, and Ras downstream pathways. CBG’s apoptotic effects were revealed through an increase in Annexin V^+^ cells in a dose-dependent manner and the activation of caspase-3. In the same context, another study reported that CBG binds to the kinase active site of EGFR, suppressing its activity and leading to apoptosis in the EGFR-overexpressing A549 and A431 cells [82]. In addition, CBG induced autophagy-dependent cell death by inhibiting EGFR-RAS pathways in human pancreatic ductal adenocarcinoma cell lines which overexpress EGFR [88]. CBG-treated HuCC-T1 and Mz-ChA-1 cholangiocarcinoma cells elevated the pro-apoptotic BAX and anti-apoptotic BCL-2 ratio and caspase-3 activation resulting in apoptosis [89].

CBG has gained interest in glioblastoma research as it is lipid-soluble which allows its translocation through the blood–brain barrier. Interestingly, CBG induced late apoptosis or necrosis in glioblastoma cells U87 (differentiated) and NIB138 (patient-derived), and glioblastoma stem cells NCH644 (differentiated) and K26 (patient-derived) with more sensitivity to cell death in glioblastoma stem cells [80]. The mechanism by which CBG induces apoptosis is mediated by the activation of caspase-3 in T98 and U87 glioblastoma cells, an effect potentiated in combination with CBD, and even further amplified upon the addition of temozolomide alongside the CBD:CBG combination [80]. Like CBN, CBG induces DNA damage and necrosis in U266B1, RPMI-8226, and SKO-007 multiple myeloma cells [83]. Notably, a study reported that CBG-treated human ovarian carcinoma A2780 (chemotherapy-sensitive) and A2780/CP70 cells (chemotherapy-resistant) showed a G0/G1 and S phase arrest and a notable increase in caspase 3/7 activity, stimulating both early and late apoptosis [68]. Similarly, CBG enhanced apoptosis in Caco-2 and HCT116 CRC cells and suppressed tumor growth in HCT116 xenografted mice [87]. Similar to CBN, CBG induced DNA damage and necrosis in U266B1, RPMI-8226, and SKO-007 multiple myeloma cells [113]. CBG also induced cell death in a spheroid 3D culture of CRC cells [121]. In addition, CBG treatment induced significant apoptosis, as evidenced by an increased BAX/BCL-2 ratio and elevated levels of caspase-3 activation in cholangiocarcinoma cells which is a rare cancer type with limited treatment options [89].

### 2.5. Cannabichromene

CBC has primarily been shown to exert anti-inflammatory, antinociceptive, and neuroprotective effects [122]. However, it may also offer therapeutic value in cancer treatment through its ability to elevate circulating levels of the endocannabinoid anandamide (AEA), which is a CB1 and CB2 agonist [123]. AEA has been shown to inhibit breast cancer cell proliferation [124] and to induce COX-2-dependent cell death in apoptosis-resistant CRC cells [125]. Recently, CBC was shown to induce necrosis as indicated by the percentage increase of PI in multiple myeloma cells and the elevation of the DNA damage marker γ-H2AX [83]. CBC exhibits significant anticancer potential due to its modulation of endocannabinoid levels, resulting in the inhibition of tumor cell proliferation and the induction of necroptosis and apoptosis.

Compared with THC and CBD, substantially less evidence is available regarding the anticancer activity of the cannabinoids CBN, CBG, and CBC. Current studies suggest that these compounds may induce different forms of cell death, including apoptosis and necrosis, depending on tumor model; however, the available evidence remains limited, and its reproducibility across experimental systems has not been established. For example, CBN has been associated with necrosis in myeloma but with apoptosis in neuroblastoma. Similarly, in addition to apoptosis and autophagy-dependent cell death, CBG has been associated with necrosis-related markers in glioblastoma models. Such heterogeneous responses may reflect differences in tumor lineage and genetic background, receptor and molecular target expression, cannabinoid concentration, and exposure conditions. Moreover, the mechanisms underlying the anticancer effects of CBN, CBG, and CBC remain considerably less characterized than those of THC and CBD. Many studies primarily report associations between cannabinoid treatment and changes in cell death-related markers, whereas relatively few have established causal mechanisms using pharmacological inhibitors, genetic approaches, or functional cell death assays. In vivo evidence also remains scarce. Therefore, although CBN, CBG, and CBC exhibit preliminary anticancer activity, their specific cell death mechanisms and therapeutic relevance require further mechanistic and in vivo validation before these findings can be generalized across tumor types.

### 2.6. Synthetic Cannabinoids

Synthetic cannabinoids are chemically engineered compounds to reproduce the effects of cannabinoids found in the cannabis plant [101]. Unlike raw cannabis extracts which contain a mixture of CB receptor agonists and antagonists, synthetic cannabinoids are single, highly pure molecules that bind with selective receptor affinity, resulting in more defined therapeutic and pharmacological activities [126]. Interestingly, diverse synthetic cannabinoids exhibit notable antitumor properties across multiple cancer types. Given the growing number of synthetic cannabinoids investigated in preclinical cancer models, this review focuses on three of the most extensively studied compounds, namely WIN55,212-2, JWH-133, and JWH-015, as representative examples with substantial evidence supporting their anticancer activity and cell death mechanisms. Although other synthetic cannabinoids, including the CB1/CB2 mixed agonist HU-210 and the CB2 receptor agonist HU-308, have also been investigated, the available evidence regarding their anticancer effects and underlying mechanisms remain comparatively limited [18,127].

WIN-55,212-2, abbreviated as WIN-55, is an aminoalkylindole derivative that acts as a potent agonist of both CB1 and CB2 receptors, unlike THC, which functions as a partial agonist at these receptors [128,129]. This synthetic cannabinoid elevated PARP and caspase-3 cleavage, led to the translocation of AIF to the nucleus, and resulted in the depletion of nicotinamide adenine dinucleotide (NAD^+^), resulting in parthanatos in HL60, MV-4-11, and KG-1a human acute myeloid leukemia (AML) cells [90]. WIN-55-induced parthanatos in AML cells was reversed through PARP1 inhibition. Furthermore, WIN-55 enhanced the antimyeloma activity of dexamethasone and melphalan bypassing resistance to melphalan in vitro, and reduced tumor growth in plasmacytoma-bearing mice [130]. WIN-55 increased BAX/BCL-2 ratio and PARP cleavage, inducing apoptosis in LNCaP prostate cancer cells [91]. It also increased the activities of caspase-3 and -9 in a dose-dependent manner and elevated oxidative stress inducing apoptosis in canine and human non-Hodgkin lymphoma cells [92]. Moreover, WIN-55 enhanced the dephosphorylation and activation of BAD causing mitochondrial depolarization and activation of the caspase cascade and apoptosis in rat C6 glioma cells [131,132].

In the same manner, JWH-133, which is a synthetic CB2-selective phytocannabinoid of the aminoalkylindole class that mimics some THC effects, decreased tumor size in B16 melanoma xenografted mice while inducing apoptosis and causing G1/S cell cycle arrest [93]. In addition, 10 μmol/L JWH-133 resulted in a 58% and 48% inhibition in the proliferation of MDA-MB231 and MDAMB468 cells, respectively, while resulting in a G0/G1 phase cell cycle arrest in MDA-MB231 cells and a significant 40% induction of apoptosis in JWH-133-treated tumors relative to control tumors [133]. Furthermore, treating the human glioma cells T98G and U87MG cells with JWH133 resulted in a decrease in their mitochondrial membrane potential, followed by a significant upregulation of the active caspase-9 and caspase-3 levels and DNA fragmentation, thereby confirming the implication of JWH-133 in inducing the mitochondrial apoptosis pathway in human glioma cells [134].

Similarly, JWH-015, a CB2 -selective agonist of the aminoalkyindole family, induced apoptosis in murine 4T1 and human MCF7 mammary carcinoma cells in a calcium-dependent manner and reduced primary tumor burden and metastasis of murine mammary carcinoma [135]. In hepatocellular carcinoma models, JWH-015 induced autophagy-dependent cell death via PPAR-γ-dependent pathways [60]. JWH-015 significantly inhibited the growth of PC-3 human prostate cancer cells by inducing CB2 receptor-mediated apoptosis. Additionally, JWH-015 resulted in G0/G1 cell cycle arrest of the PC-3 cells, reduced Akt signaling and lead to the de novo synthesis of ceramide, thus the activation of the ceramide/JNK pathway. Finally, JWH-015 significantly decreased the tumor volume and weight of prostate cancer xenografts in nude mice compared with the control group [136]. The mechanisms by which the previously covered synthetic cannabinoids trigger cell death in different cancer models are summarized in Table 1.

The heterogeneous effects of synthetic cannabinoids further illustrate how the pharmacological characteristics of individual compounds can influence cannabinoid-induced cell death. Compared with phytocannabinoids, many synthetic cannabinoids exhibit more clearly defined receptor specificity and selectivity, thereby delineating the mechanistic pathways and their biological outcomes. This high agonist specificity and efficacy, receptor abundance, and downstream signaling contribute to the variable responses observed across compounds and tumor models. Importantly, the defined pharmacological profiles of certain synthetic cannabinoids enable more rigorous mechanistic interrogation through receptor antagonism, pathway inhibition, and genetic approaches, providing stronger evidence for causal relationships than the assessment of cell death-associated markers alone. Nevertheless, most available evidence remains preclinical, and differences in receptor pharmacology, experimental models, concentrations, and treatment conditions limit direct comparisons among synthetic cannabinoids. Future studies should establish pharmacologically relevant concentrations and exposure durations, define receptor-dependent and receptor-independent signaling cascades, and validate the proposed mechanisms using complementary genetic and pharmacological approaches and appropriate in vivo models. Such studies will be essential to determine the translational potential of synthetic cannabinoids as anticancer agents.

## 3. Cannabinoids Combinations in Cancer Cell Death

The rational for combining phytocannabinoids in inducing cancer cell death is due to their ability to activate multiple receptors and to trigger several signaling pathways. This multifaceted function of cannabinoids enhances their therapeutic outcomes in complex diseases such as cancer [137].

### 3.1. THC-CBD Combinations

Combining the psychoactive compound THC (mostly Δ^9^-THC) with non-psychoactive phytocannabinoids allows the use of lower THC doses, potentially reducing its psychoactive side effects in clinical applications. Interestingly, botanical preparations containing THC were found to display greater cytotoxicity compared to pure cannabinoids [138,139]. For instance, treatment with submaximal doses of THC and CBD in a 1:1 ratio significantly decreased the viability of various human glioma cell lines and primary cultures [59]. The combined administration of submaximal doses of THC (7.5 mg/kg/day) and CBD (7.5 mg/kg/day) more effectively inhibited the growth of U87MG cell-derived subcutaneous xenografts than either each agent alone and exhibited a comparable effect to a higher, effective dose of THC (15 mg/kg/day) [59]. Consistently, a 1:1 combination of THC and CBD disrupted mitochondrial respiration, induced mitochondrial morphological changes, triggered cytochrome c release into the cytosol, and reduced the expression of electron transport chain subunits, including complexes I and IV [140]. These effects were mediated by the activation of CB1, CB2, and TRPV1 receptors, and autophagic degradation. This suggests that the CBD-THC combination may activate additional pathways, which warrants deeper investigation. Furthermore, the administration of a Sativex-like preparation, which is a laboratory preparation comprising equal amounts of THC-CBD, in in vitro and in vivo melanoma models significantly impaired tumor growth and induced TRIB3-dependent, noncanonical autophagy-dependent cell death-mediated apoptosis through ATG-7-dependent pathways [55].

Additionally, a 3:1 THC-CBD combination in MDA-MB-231 and MCF-7 breast cancer cell lines, resulted in phosphatidylserine translocation to the outer cytoplasmic membrane, DNA fragmentation, and activation of mitochondrial and caspase-dependent apoptosis pathways [55,57]. Interestingly, a 3:1 THC-CBD mixture led to the activation of mitochondrial and caspase-dependent apoptosis in MDA-MB-231 and MCF-7 cells. Mechanistically, the cannabinoid mixture induced a dose- and time-dependent increase in total apoptosis as determined by Annexin V/PI dual staining flow cytometry. In addition, a significant increase in DNA fragmentation was detected by ELISA, accompanied by elevated levels of PARP cleavage. At the highest treatment concentration (17.5 µg/mL), cleaved PARP protein levels increased approximately 2- and 3.5-fold in MDA-MB-231 and MCF-7 cells, respectively. Finally, the BAX/BCL-2 ratio significantly increased in a dose-dependent manner in both cell lines, accompanied by an elevated release of cytochrome c into the cytoplasm [57]. In parallel, combining THC and CBD lead to synergistic antiproliferative effects in HeLa and SiHa cervical cancer cells. Compared with either cannabinoid alone, the combination exerted more significant reductions in cell viability and enhanced apoptosis and autophagy-dependent cell death activation, while disrupting cell cycle progression [141]. Collectively, these results suggest that co-treatments with THC and CBD exert a more pronounced inhibitory effect on the proliferation and survival of cancer cells compared with single treatments.

### 3.2. THC-CBC Combinations

A synergistic interaction between Δ^9^-THC and CBC was observed using the urothelial cell carcinoma cell lines T24 and HTB-9 [58]. The combination treatment of THC-CBC (2.8 + 17.2 μg/mL) resulted in 76% apoptosis in T24 cells compared to 32% in the control [58]. The proposed mechanism involved CB2 receptor-mediated activation of caspase-3 and ceramide-dependent apoptosis of bladder cancer cells. Additionally, the induction of apoptosis was associated with the downregulation of AKT phosphorylation and the activation of TRPV2 channels.

### 3.3. Other Cannabinoid Combinations

In ovarian cancer cells, the most potent synergistic phytocannabinoid combinations were THC-CBC and THC-CBC-CBG formulated at ratios of 7.5:2.5 and 7.4:2.5:0.1, respectively [81]. In SW-620 CRC cells, treatment with 1.5 µg/mL or 3 µg/mL of CBD-CBG combination resulted in a greater reduction in cell viability than single treatments [121]. Furthermore, in U87, U373, and T98 glioblastoma cell lines, treatment with the CBD-CBG combination at IC_50_ concentrations induced the most pronounced activation of caspase-3 [80].

Combination studies reported enhanced anticancer activity compared with individual cannabinoids. The magnitude of those effects appears to depend not only on tumor origin and cannabinoid concentration, but also on the specific compounds and their relative ratios. Differences in cannabinoid ratios may alter receptor engagement and downstream signaling, potentially influencing oxidative stress, mitochondrial dysfunction, ER stress, and the relative contributions of distinct cell death mechanisms. However, the molecular basis underlying these combination effects remains incompletely characterized and requires further mechanistic investigation.

The translational relevance of these findings is currently limited by the scarcity of in vivo studies. Although selected cannabinoid combinations have demonstrated enhanced suppression of tumor growth in xenograft models, the doses, exposure conditions, and treatment schedules used experimentally may not adequately reflect clinically achievable exposures. Moreover, most combination studies have focused on short-term effects and cell death-associated markers with limited assessment of long-term tumor control, systemic toxicity, pharmacokinetics, or the emergence of therapeutic resistance. Further studies incorporating clinically relevant dosing strategies, pharmacokinetic and pharmacodynamic characterization, long-term efficacy and safety assessments, and genetically diverse tumor models are, therefore, required to determine whether the observed combination effects can be translated into effective therapeutic strategies. recommendations.

### 3.4. Cannabinoid Combinations with Anticancer Therapies

Cannabinoids have been shown to synergize with and enhance the efficacy of various conventional anticancer therapies, thereby sensitizing cancer cells, particularly therapy-resistant ones, to therapies across diverse cancer types including CRC, osteosarcoma, glioma, glioblastoma, multiple myeloma, leukemia, triple-negative breast cancer, as well as pancreatic, bladder, liver, and cervical cancers. This combinatorial approach may assist in overcoming therapeutic resistance, which is considered one of the major challenges in oncology.

For instance, when administered in combination in preclinical studies, cannabinoids can enhance the efficacy of various commonly used chemotherapeutics when used in combination such as 5-fluorouracil [142,143], doxorubicin [144,145,146,147,148,149], gemcitabine, and cisplatin [141,150,151,152], temozolomide [59,153], dasatinib [154], nivolumab [155], carfilzomib [156], and anti-leukemia drugs [157,158].

In addition, they have been reported to synergize with photodynamic therapy [159,160,161,162,163], radiation [164,165,166], oxygen/ozone [167,168], platinum drugs [169], carbon monoxide [170], and other natural compounds [171,172].

Conversely, evidence of combining cannabinoids with immunotherapy remain complex and, at times, contradictory. For instance, a retrospective observational study evaluated the effect of cannabis use during immunotherapy treatment using nivolumab on the response rate, progression-free survival, and overall survival in patients with advanced melanoma, non-small cell lung cancer, and renal clear cell carcinoma. Results showed that cannabis use significantly reduced the response rate to immunotherapy without affecting the progression-free and overall survival [155]. Another retrospective cohort study confirmed an antagonistic interaction between cannabis and immunotherapy since the consumption of cannabis worsened the overall and progression-free survival as well as the disease control rate in cancer patients with solid malignancies and receiving immunotherapy [173]. On the other hand, substantial clinical evidence suggest that cannabinoids can effectively alleviate several cancer- and treatment-related symptoms such as pain, nausea, anorexia, and anxiety, and sleep disturbances, thereby enhancing the quality of life of many cancer patients [174,175,176]. Knowing that the undesired cannabinoid-induced immunosuppressive effects are mainly mediated through CB2 receptor signaling, a study proposed that CB2 receptor neutralization during cannabinoid treatment may preserve the benefits of cannabinoids while minimizing their potential interference with antitumor immune responses. In this context, since CBD and CBG have little agonism with CB2 receptors, these two phytocannabinoids can be considered better treatment options alongside immunotherapy [177]. For instance, it has been shown that although CBD elevated the proportion of CD19-CAR T cells in the sub-G1 phase in the cell cycle, the antitumor effect and cytokine secretion of those cells were not affected by CBD treatment in hematologic malignancies [178]. Collectively, these findings emphasize that although some cannabinoids can be effective in the management of cancer- and treatment-related symptoms, they also exert significant immunomodulatory effects and may influence antitumor responses. These observations highlight the need for careful consideration during the usage of cannabinoids in cancer patients in general and in patients receiving immunotherapy in specific.

Despite increasing interest in cannabinoid-based combination approaches, evidence investigating their combination with targeted anticancer therapies remains relatively limited, particularly at the clinical level. The combination between cannabis and the multikinase inhibitor Sorafenib consistently activated several stress- and inflammation-related pathways, including TNF/NF-κB, MAPK, JAK–STAT, oxidative stress, and p53-mediated apoptosis, together with the suppression of metabolic and proliferative mechanisms [179]. In addition, the combination of CBD and the PARP inhibitor Olaparib resulted in an increase in apoptosis and G2/M phase arrest in breast cancer cells [180]. The combination of CBD and the multikinase inhibitor cabozantinib elevated apoptosis in hepatocellular carcinoma cells while inducing ER stress and subsequently activating the p53 pathway [181]. Finally, a synergistic interaction was observed in ovarian cancer cells upon the combination of THC, CBC, CBG, and the PARP inhibitor niraparib, thereby increasing cytotoxicity observed in these cells. Interestingly, combining a cannabis mix containing THC, CBG, CBN, CBC, with niraparib resulted in substantial cell death of patient’s cells isolated from a cancerous deep femoral lymph node [81].

Taken together, the available evidence on cannabinoids combinations emphasizes the importance of further research which should address the pharmacological interactions between cannabinoids and standard cancer treatments and compounds to establish safe and effective combination therapies [101]. In addition, most of these combination studies between cannabinoids and other compounds or anticancer therapies have been demonstrated in cancer cell lines, highlighting the need for validation in animal models, and ultimately in future clinical studies.

Beyond synergizing with chemo- and radiotherapy, cannabinoids show promise in palliative care, alleviating cancer-related symptoms and the side effects of conventional cancer treatments [182,183]. For instance, THC and CBD, whether administered alone or in combination, effectively reduced chemotherapy-induced nausea and vomiting, a common adverse effect [14,184]. CBD significantly alleviated cancer-related depression, anxiety, and sleep disorders, such as insomnia [185]. About 20% of cancer-related deaths are attributed to metabolic alterations and weight loss, known as cachexia [186]. Several studies reported that THC and CBD, administered alone and in combination, efficiently improve appetite and caloric consumption, in addition to reducing insulin resistance, weight loss, and lipid storage depletions [187]. These findings highlight the efficiency of phytocannabinoids in mitigating classical chemotherapy side effects, enabling cancer patients to better tolerate intensive treatment regimens. Interestingly, several ongoing clinical trials are investigating cannabis for managing cancer symptoms and treatment side effects. These include observational studies in adults and pediatric patients assessing cannabis/CBD use for pain, seizures, sleep, mood, and treatment-related symptoms [188,189]. Moreover, an ongoing phase II interventional clinical trial is evaluating the efficacy of three cannabis extract combinations namely, high THC-low CBD, low THC-high CBD, or equal amounts of THC and CBD against a placebo for alleviating cancer-related symptoms [189].

## 4. Clinical Evidence on Cannabinoids in Cancer

Despite the increasing flux of preclinical evidence highlighting diverse cannabinoids as effective anticancer agents and investigating their anticancer potential, few clinical evidence supporting these antitumor activities is present. In fact, most of the ongoing and completed clinical trials registered on ClinicalTrails.gov focus on the medical use of cannabinoids in the management of cancer- and/or cancer treatment-related symptoms including nausea, vomiting, appetite, pain, anxiety, and sleep as well as the enhancement of the quality of life and well-being of cancer patients, especially those receiving palliative care. Most of those clinical trials are randomized interventional phase I or phase II mainly assessing diverse THC, CBD, or THC and CBD-based interventions [174,176,190,191,192,193,194,195,196,197]. Fewer clinical trials focused on studying other phytocannabinoids like CBC and CBG [198,199]. Furthermore, some clinical trials focus on assessing the overall safety and tolerability of cannabinoids [176,196,200,201]. For instance, an interventional phase I trial assessed the safety and tolerability of daily oral cannabinoids administration (5 mg THC, 15 mg THC, or 15 mg THC combined with 15 mg CBD) compared with placebo in patients receiving active anticancer therapy [202].

In parallel, much fewer clinical trials focus on investigating the efficacy of cannabinoids as anticancer agents. For instance, a randomized phase II interventional trial, known as the ARISTOCRAT trial, aims to compare the efficacy of combining nabiximols with the chemotherapeutic agent temozolomide versus a matched placebo in patients with recurrent O6-methylguanine-DNA methyltransferase methylated glioblastoma [203]. In addition, an ongoing interventional phase II clinical trial aims at investigating the antitumor effect of the administrations of cannabis oil consisting of 10% THC and 5% CBD three times daily in patients with advanced untreatable hepatocellular carcinoma with primary objectives of assessing tumor size and tumor markers α-fetoprotein and des- γ -carboxyprothrombin [204]. Furthermore, an ongoing interventional phase I clinical trial aims at investigating the anticancer efficacy of an oral administration of “CBD high dose” and “CBD low dose” interventions on patients with breast cancer in comparison to low and high dose placebo. The antitumor effects of those interventions will be assessed by measuring Ki67 expression as a marker reflecting possible biological changes in relation to cell proliferation and apoptosis in the primary tumors of the breast cancer patients [205].

Numerous limitations persist despite these clinical trials. First, large-scale randomized clinical trials are still lacking as most research on cannabinoids comprises small cohorts or preclinical models, restricting robustness of evidence supporting the efficient use of cannabinoids in cancer therapy, especially with the long-term usage. Second, most research focuses on short-term outcomes, leaving uncertainty around the chronic effects thereby hindering the evaluation of the risk–benefit ratio of prolonged cannabinoid use. Third, a major limitation is the lack of standardized cannabinoid formulations and dosing protocols. Clinical trials assess widely varying ratios of individual cannabinoids, cannabinoid-based pharmaceuticals, or cannabis extracts often without specifying a robust pharmacological rationale for the selected dosages or compositions. In addition, the quality and purity of different cannabinoid-based interventions are not well-characterized, and the used cannabinoid preparations may vary in compound concentrations, contaminant levels, and extraction methods. This heterogeneity hinders the development of standardized therapeutic protocols, limits comparisons and interpretations of outcomes across different studies, and reduces reproducibility across studies, reinforcing the need for developing robust protocols to ensure the consistency and quality of cannabinoid-based therapies. Fourth, although the pharmacokinetics (absorption, distribution, metabolism, and excretion) of THC and CBD have been extensively reviewed [206], the application of those parameters in clinical trials as well as a comprehensive research on the pharmacodynamics (biological effects and mechanisms of action) of cannabinoids in cancer patients is still lacking [101]. Unfortunately, an interventional phase I and II clinical trial pharmacokinetic, pharmacodynamic, safety and stability evaluation of three formulations of THC (5 mg/mL of THC oral solution, THC: CBG 5 mg/mL each as oral solution, and THC: CBC 5 mg/mL each as oral solution) in healthy versus post-chemotherapy patients is listed as having an unknown status on ClinicalTrials.gov. [207]. As a result of these limitations, robust clinical evidence supporting cannabinoids’ anticancer effects obtained in preclinical studies is still lacking.

To address the current uncertainties, well-designed, large-scale, and long-term clinical and pharmacological studies are needed to establish the safety and tolerability of cannabinoids or cannabinoid-based interventions in cancer patients whether alone or in combination with other pain management or cancer therapies. In parallel, comprehensive investigations on the pharmacodynamics and pharmacokinetics of those interventions are essential to optimize and standardize cannabinoid formulations, compositions, and dosage protocols, thereby ensuring homogeneity and comparability across different clinical trials. Such standardization will ultimately enable more robust evaluations of the therapeutic potential and underlying molecular mechanism of action of those cannabinoid-based interventions in oncology, ensuring their translation into evidence-based practice.

## 5. FDA-Approved Cannabinoid-Based Pharmaceuticals

Currently, there are four cannabis-based pharmaceutical preparations approved by the USA FDA namely, Epidiolex^®^, Marinol^®^, Syndros^®^, and Cesamet^®^ [208]. Epidiolex^®^ is a purified plant-derived CBD oral anticonvulsant solution indicated for seizures associated with Lennox–Gastaut syndrome or Dravet syndrome in patients two years of age and older. Marinol^®^ and Syndros^®^ both contain dronabinol, a synthetic form of Δ^9^-THC; Marinol is formulated as capsules, while Syndros is an oral solution—both are approved for acquired immunodeficiency syndrome AIDS-related anorexia and chemotherapy-induced nausea and vomiting unresponsive to standard antiemetics. Cesamet^®^ (nabilone) is a synthetic cannabinoid analogue used for chemotherapy-induced nausea/vomiting refractory to other treatments and is the only structural modification of Δ^9^-THC that has led to an approved drug [209]. In contrast, Sativex^®^ (nabiximols), an oromucosal spray containing a 1:1 THC:CBD ratio, has been approved across the European Union and Canada for the management of moderate-to-severe multiple sclerosis-related spasticity unresponsive to first-line treatments, but it is not yet FDA-approved.

## 6. Barriers to the Clinical Translation of Cannabinoid-Based Therapies

While the research unraveling the promising anticancer effects of cannabinoids in inducing regulated cell death and suppressing tumorigenesis is continuously growing, their medical use remains limited due to several factors.

One crucial limitation for the use of cannabinoid-based therapies is their safety profile and abuse potential, particularly that of THC, which is known as the primary psychoactive constituent of the *Cannabis sativa*. THC exerts its psychoactive effects primarily through activation of central cannabinoid CB1 receptors and has been linked with paranoia, impaired memory and attention, anxiety, euphoria, and transient psychotic symptoms [210]. Thus, safety concerns arise regarding the physical dependence, abuse, and intolerance to THC due to the repeated or long-term exposure especially at high doses [211]. Consequently, the psychoactive and abuse-related potential of THC remains an important barrier to the clinical translation of preclinical data into successful clinical trials, thereby emphasizing the need for safety and tolerability studies along with standardized dosing and composition protocols. Interestingly, combining THC with CBD may mitigate THC’s psychotropic effects, but outcomes depend on factors such as THC:CBD ratio, dosage, administration route, patient history, treatment duration, and timing of cannabinoid administration.

Furthermore, the restricted legalization and prevailing societal stigma surrounding medical cannabinoids in many countries, including the MENA region, have limited research progress exploring their therapeutic potential. This substantially hindered the feasibility of conducting large-scale clinical trials and the development of standardized cannabinoid-based therapies. However, as scientific knowledge advances, public perceptions change, and policymakers adapt to support the medical use of cannabinoids, there are hopes that these laws and regulations can be prone to change.

Another essential challenge limiting the clinical translation of cannabinoids in oncology is the marked heterogeneity of human tumors. Distinctions in genetic, epigenetic, and molecular characteristics among and within tumors may affect the expression of cannabinoid receptors, responsiveness to cannabinoid-based therapies, and the underlying signaling and molecular pathways rendering therapeutic efficacy largely context- and tumor-dependent [212].

Moreover, drug–drug interactions may occur between cannabinoids and anticancer agents when administered simultaneously. For instance, such interactions may occur since THC and CBD are metabolized by, and may inhibit, cytochrome P450 enzymes and drug transporters associated with the pharmacokinetics of several anticancer therapies, thereby influencing drug exposure, therapeutic efficacy, and toxicity [206,213,214].

Finally, a major barrier against precision and effective cannabinoid-based therapies is the lack of validated and clinically established predictive biomarkers. These limitations prevent the opportunity of identifying therapeutic response and patient populations or tumor types that would most likely benefit from the corresponding cannabinoid-based therapy. Furthermore, despite the substantial preclinical studies supporting the use of cannabinoids as anticancer agents modulating diverse cell death mechanisms, their underlying molecular mechanisms remain only partially described. This partial mechanistic understanding further limits the optimization and implementation of standardized dosages and compositions of cannabinoid-based interventions.

## 7. Discussion

**Figure 2:**
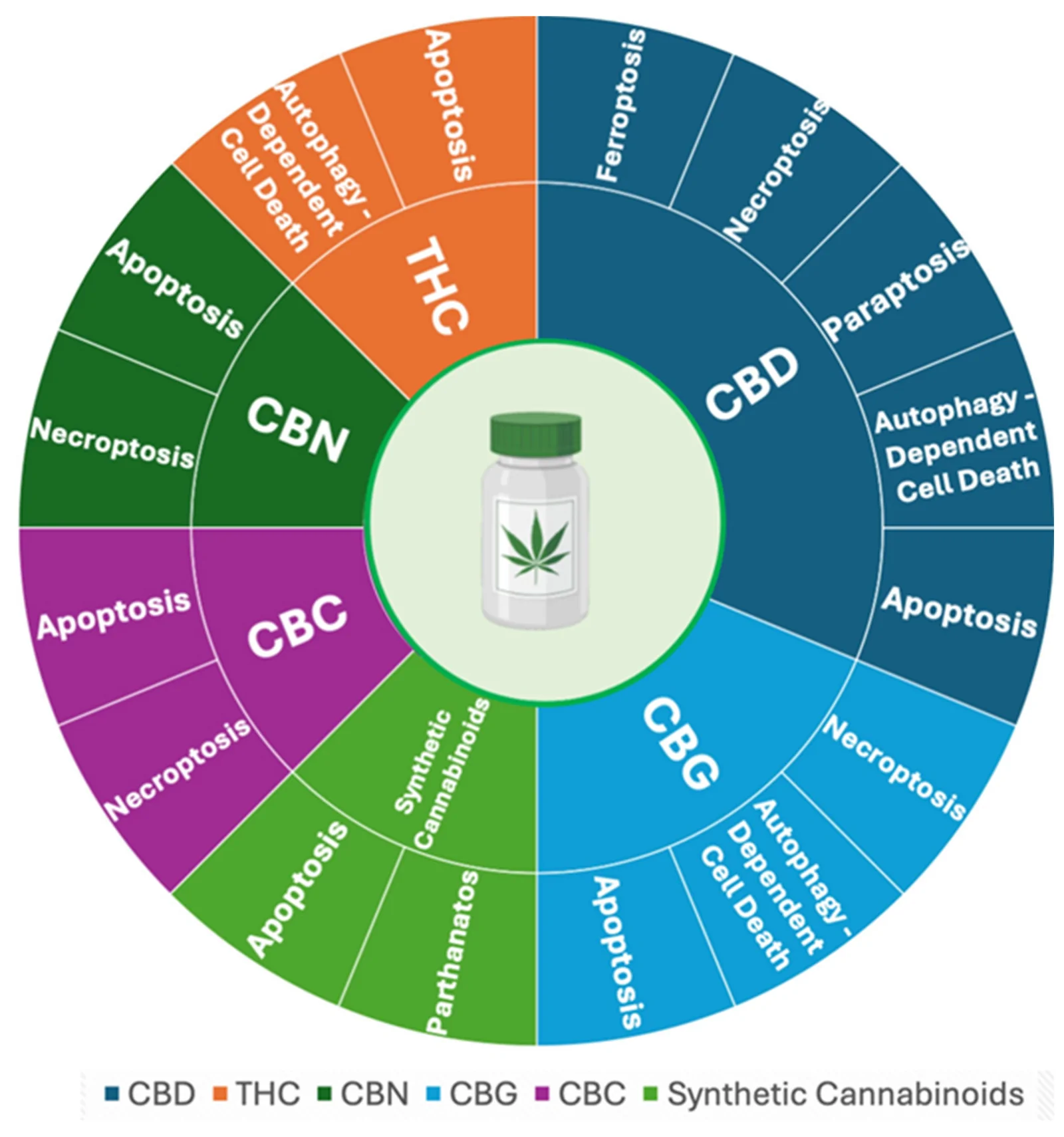
Cannabinoid-regulated cell death modalities in cancer. *CBC* cannabichromene; *CBD* cannabidiol; *CBG* cannabigerol; *CBN* cannabinol; *THC* tetrahydrocannabinol.

In this review, we highlighted the therapeutic promise of cannabinoids in cancer and emphasized their ability to modulate diverse cell death mechanisms in multiple tumor types (Figure 2). Evading cell death is one of the critical hallmarks of cancer, therefore the ability to trigger cell death pathways remains a central strategy in the development of anticancer therapies [23].

Cannabinoids, whether endogenous (2-AG, AEA), plant-derived (THC, CBD, CBN, CBG, CBC), or synthetic (WIN-55, JWH-133, JWH-015), can modulate a broad spectrum of signaling pathways, including PI3K/AKT/mTOR, MAPK/ERK, oxidative stress, and ceramide synthesis. Through these interactions, cannabinoids elicit antitumor responses such as the inhibition of cancer cell proliferation, and the induction of cell death, angiogenesis, metastasis, and stemness, among other key players of tumorigenesis. Cannabinoids predominantly induced cell death through apoptosis and autophagy-dependent cell death. Nevertheless, other cell death mechanisms including necroptosis, ferroptosis, parthanatos, and pyroptosis, have also been reported. However, these responses are neither exclusive to individual cannabinoids nor consistent across experimental models. The cellular response to a given cannabinoid may depend on tumor type, receptor and molecular target expression, cannabinoid concentration, exposure duration, treatment conditions, and tumor genetic background. For example, CBD induces apoptosis in several cancer models but has also been associated with autophagy-dependent cell death, ferroptosis, and lethal mitophagy in glioblastoma models. Furthermore, different responses to CBD have been reported across breast cancer subtypes. In MCF7 cells, but not in MDA-MB-231, CBD induces cellular stress, whereas other studies in TNBC have implicated CBD in the modulation of autophagy-dependent cell death. These observations emphasize that assigning a specific cell death modality to a cannabinoid requires more than demonstrating reduced cell viability or changes in cell death-associated readouts, such as Annexin V positivity or LC3-II accumulation. Instead, functional and mechanistic studies are required to establish the causal involvement of the molecular machinery responsible for the observed cell death phenotype.

Despite differences among tumor models and cannabinoid compounds, several recurring responses have emerged, including reduced cell viability, caspase activation, oxidative stress, mitochondrial dysfunction, and alterations in autophagy-dependent cell death processes. However, substantially fewer studies have delineated the complete signaling cascade linking cannabinoid engagement of specific receptors or molecular targets to downstream signaling events and the execution of a defined cell death program. Greater use of pathway-specific pharmacological inhibitors or activators, receptor antagonists, genetic knockdown or knockout approaches, and functional rescue experiments is needed to distinguish causal mechanisms from associations with cell death-related markers.

In vitro cancer models provide well-controlled experimental system for delineating receptor signaling, concentration-response relationships, and molecular mechanisms of cannabinoid action. Nevertheless, these models cannot fully recapitulate the complex tumor microenvironment, including interactions with immune and stromal cells, extracellular matrix components, hypoxia, vascularization, cannabinoid metabolism, and pharmacokinetics. Animal studies have provided important evidence supporting the antitumor activity of several cannabinoids but remain substantially less extensive than in vitro investigations. Moreover, the frequent use of xenograft models in immunodeficient mice limits assessment of the contribution of the immune system to cannabinoid-mediated antitumor responses. The incorporation of immunocompetent, syngeneic, genetically engineered, and other physiologically relevant tumor models could, therefore, provide a more comprehensive understanding of cannabinoid activity within the tumor microenvironment.

Collectively, phytocannabinoids and synthetic cannabinoids differ substantially in their receptor selectivity, downstream signaling, cell death mechanisms induction, cancer models investigated, and levels of preclinical evidence and clinical validation. These similarities and distinctions are summarized in Table 2. In addition, phytocannabinoids exhibit distinct pharmacokinetic profiles which may also vary not only among individual compounds but also according to the route of administration (Table 3). In contrast, the pharmacokinetic profiles of synthetic cannabinoids remain poorly characterized, with limited data available. Interestingly, combining multiple cannabinoids has been shown to enhance their antitumor efficacy compared to individual compounds, potentially through synergistic interactions that amplify pro-death signaling while allowing the use of lower doses of psychoactive components such as THC, thereby reducing side effects. Furthermore, a growing body of evidence is also supporting the use of cannabinoids in combination with conventional anticancer therapies, whereby they have shown the potential to improve therapeutic efficacy, sensitize resistant cancer cells, and enhance treatment responses across diverse tumor types. However, these interactions remain highly context-dependent, particularly in immunotherapy, confirming the need for further mechanistic and clinical investigations.

Notably, some studies have also demonstrated the anticancer potential of cannabis extracts and oils against various cancer types. For instance, Lebanese cannabis oil extract induced both extrinsic and intrinsic apoptosis-independent of ROS production- of acute myeloid leukemia using WEHI-3 cells by inhibiting MAPK/ ERK pathway [240]. Moreover, cell death is also induced when using cannabis extracts and/or oils in prostate cancer [109], melanoma [241], pancreatic cancer [242], glioblastoma [243], CRC [244], hepatocellular carcinoma [245], as well as other cancer types. Of note, other minor phytocannabinoids, not addressed in this review, can also exert some significant antitumor effects such as cannabidivarin, cannabicyclol, and cannabigerovarin [83,246].

In this review, we emphasized that clinical translational studies remain limited due to the inability to use cannabinoids in large, randomized trials, insufficient information regarding the consequences of prolonged treatment, and incomplete characterization of their pharmacokinetic and pharmacodynamic profiles in patients with cancer. Future in vitro studies should prioritize rigorous mechanistic validation of cannabinoid-induced cell death, whereas in vivo investigations should establish dose–response relationships, clinically relevant exposure schedules, therapeutic windows, systemic toxicity, and pharmacokinetic–pharmacodynamic relationships. In parallel, formulation and drug-delivery strategies aimed at improving cannabinoid bioavailability and compositions, exposure at tumor sites, and pharmacokinetic properties may help overcome current therapeutic limitations. Ultimately, integrating mechanistically robust cellular studies with physiologically relevant animal models and well-designed clinical investigations will be essential to determine whether cannabinoid-mediated cell death can be translated into safe and effective anticancer strategies. Finally, although multiple cannabinoid-based pharmaceuticals have received regulatory approval, their clinical use in oncology remains primarily limited to the management of cancer- and treatment-related symptoms. Their use in diseases and syndromes other than cancer is also more established than their application as direct anticancer therapies, highlighting the need for additional clinical evidence supporting their use as antitumor agents.

## 8. Conclusions

**Figure 3:**
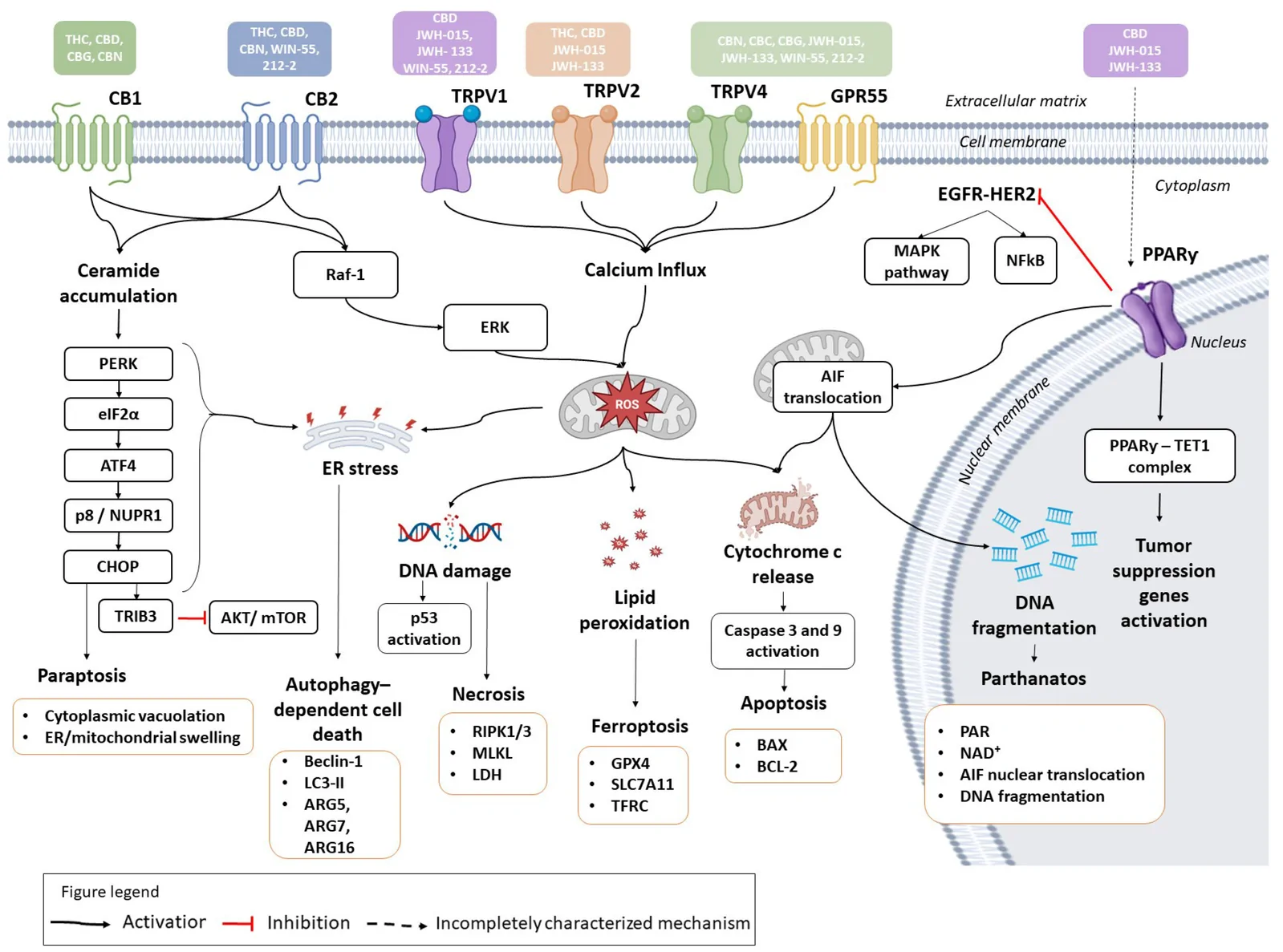
Schematic illustration of the molecular pathways and mechanisms underlying the antitumor effects of cannabinoids. AIF Apoptosis Inducing Factor; AKT Protein Kinase B; *ARG* autophagy-related genes; ATF4 activating transcription factor; BAX Bcl-2 associated X protein; BCL-2 B-cell lymphoma 2; CBC cannabichromene; CBD cannabidiol; CBG cannabigerol; CBN cannabidiol; CHOP C/EBP homologous protein; DNA deoxyribonucleic acid; eIF2α eukaryotic initiation factor 2 alpha; ER endoplasmic reticulum; ERK extracellular signaling regulated kinase; GPR55 G protein coupled receptor 55; GPX4 glutathione peroxidase 4; HER2 human epidermal growth factor receptor 2; LC3-II microtubule associated protein 1 light chain 3-II; LDH lactate dehydrogenase; MAPK mitogen activated protein kinase; MLKL mixed linage kinase domain like protein; mTOR mechanistic target of rapamycin; NAD+ nicotinamide adenine dinucleotide; NFkB nuclear factor kappa B; NUPR1 nuclear protein 1; p53 tumor protein p53; PAR Poly(ADP-ribose); PERK protein kinase R-like endoplasmic reticulum kinase; PPARy peroxisome proliferator activated receptor gamma; Raf-1 seine/threonine kinase; RIPK1 receptor-interacting protein kinase 1; RIPK3 receptor-interacting protein kinase 3; ROS reactive oxygen species; SLC7A solute carrier family 7 member A; TFRC transferrin receptor protein 1; THC tetrahydrocannabinol; TRIB3 tribbles pseudokinase 3; TRPV1 transient receptor potential vanilloid 1; TRPV2 transient receptor potential vanilloid 2; TRPV4 transient receptor potential vanilloid 4.

In summary, this review highlights the growing body of evidence supporting the ability of cannabinoids to induce multiple forms of cell death across a broad spectrum of cancer types (Figure 2). Through the modulation of diverse molecular and signaling pathways, cannabinoids have emerged as promising anticancer agents, mostly in preclinical models, with the potential to target tumor growth, survival, and progression, ultimately triggering diverse cell death mechanisms (Figure 3). However, significant gaps in knowledge remain regarding the therapeutic efficacy, safety, and mechanistic complexity of cannabinoids, in particular in the clinical setting.

Further research is necessary to understand and improve cannabinoids therapeutic potential across different tumor types. It remains to be determined whether additional cannabinoids can induce novel forms of cell death in cancer preclinical models and in patients or exhibit enhanced antitumor activity in different cancer settings. Importantly, rigorous preclinical investigations and well-designed clinical trials are required to validate these findings, elucidate the intricate signaling networks involved, and optimize cannabinoid-based therapeutic strategies. Collectively, continued advances in this field may pave the way for the development of novel cannabinoids-based cancer therapies that complement or enhance current cancer treatment approaches.

## Figures and Tables

**Table 2:** Comparative characteristics of phytocannabinoids and synthetic cannabinoids in cancer research. 5-HT1A 5-hydroxytryptamine receptor 1A; AKT/mTOR protein kinase B/mammalian target of rapamycin; ATF4 activating transcription factor 4; CB1 cannabinoid receptor 1; CB2 cannabinoid receptor 2; CBC cannabichromene; CBD cannabidiol; CBG cannabigerol; CBN cannabinol; CHOP C/EBP homologous protein; EGFR epidermal growth factor receptor; eIF2α eukaryotic initiation factor 2 alpha; ER endoplasmic reticulum; ERK extracellular signal-regulated kinase; GPR55 G protein-coupled receptor 55; JNK c-Jun N-terminal kinase; JWH015 (2-methyl-1-propyl-1H-indol-3-yl)-1-naphthalenylmethanone; JWH133 (6aR,10aR)-3-(1,1-dimethylbutyl)-6a,7,10,10a-tetrahydro-6,6,9-trimethyl-6H-dibenzo[b,d]pyran; MAPK mitogen-activated protein kinase; MAPK/ERK mitogen-activated protein kinase/extracellular signal-regulated kinase; MAPK/JNK mitogen-activated protein kinase/c-Jun N-terminal kinase; NF-κB nuclear factor kappa B; p8 nuclear protein 1; p38 p38 mitogen-activated protein kinase; PERK protein kinase RNA-like endoplasmic reticulum kinase; PERK-eIF2α-ATF4-CHOP protein kinase RNA-like endoplasmic reticulum kinase/eukaryotic initiation factor 2 alpha/activating transcription factor 4/C/EBP homologous protein; PI3K/Akt phosphoinositide 3-kinase/protein kinase B; PI3K/AKT/mTOR phosphoinositide 3-kinase/protein kinase B/mammalian target of rapamycin; PPARγ peroxisome proliferator-activated receptor gamma; Raf1/ERK Raf-1/extracellular signal-regulated kinase; THC Δ9-tetrahydrocannabinol; TNBC triple-negative breast cancer; TRPA1 transient receptor potential ankyrin 1; TRPM8 transient receptor potential melastatin 8; TRPV transient receptor potential vanilloid; WIN55 WIN55,212-2 (R)-(+)-[2,3-dihydro-5-methyl-3-(4-morpholinylmethyl)pyrrolo [1,2,3-de]-1,4-benzoxazin-6-yl]-1-naphthalenylmethanone mesylate. → leads to.

Cannabinoid	Receptor Affinity	Principle Signaling Pathways	Predominant Modes of Cell Death	Cancer Types Investigated	Level of Experimental Evidence
THC	Partial agonist at CB1 and CB2 [209] Agonist at GPR55 and TRPVs, and to some extent PPARγ [41]	p8 function through PERK-eIF2α-ATF4-CHOP → mitochondrial -mediated cell death [50] ER stress, oxidative stress → Raf1/ERK activation [51] Ceramide-mediated ER stress → p8 → AKT/mTOR activation [53]	Apoptosis Autophagy	Solid and liquid cancers including endometrial cancer, oral cancer, colorectal cancer, melanoma, astrocytoma, glioma, and leukemia	Anticancer evidence: available preclinical evidence, very limited clinical evidence Supportive-care evidence: available clinical evidence
CBD	At very low (nanomolar to micromolar) concentrations Antagonist at GPR55 [215] Antagonist at TRPM8 channel [215] Agonist at PPAR-γ and TRPV1 TRPV2 channels [216] Noncompetitive CB1 receptor antagonist. Inverse agonist at the CB2 receptor [217] Agonist at 5-HT1A [218]	PI3K/AKT/mTOR, MAPK/ERK, NF-κB [100,103] JNK, p38, and ERK pathways [65]	Apoptosis Autophagy Ferroptosis To less extent: paraptosis, necrosis, mitophagy, and macroautophagy	Solid and liquid cancers including breast cancer/TNBC, melanoma, glioma, glioblastoma, head and neck squamous cell carcinoma, oral squamous carcinoma, endometrial, ovarian, cervical, colorectal, lung, gastric, prostate cancer, and leukemia.	Anticancer evidence: available preclinical evidence, very limited clinical evidence Supportive-care evidence: available clinical evidence
CBN	Agonist action at both CB1 and CB2 [219] Activates TRPA1 [123] Inhibits TRPM8 [123] Ineffective at TRPV1 [123] Inhibits TRPV2 [123] Limited agonist at TRPV3 and TRPV4 [220]	AKT pathway	Apoptosis To a less extent, necrosis	Solid and liquid cancers including multiple myeloma, neuroblastoma, glioma, hepatocellular carcinoma, lung adenocarcinoma, cholangiocarcinoma, epidermoid cancer, breast cancer and acute myeloid leukemia.	Anticancer evidence: less available preclinical evidence, very limited clinical evidence Supportive-care evidence: less available clinical evidence
CBG	Low affinity for CB1 and CB2 [221] Weak agonist at TRPV1 and TRPV2 [123] Potent antagnonist at TRPM8 [123] Potent agonist at TRPA1 [123] Antagonist at 5-HT1A [222] Activates both PPARα and PPARγ in vitro [223]	EGFR, AKT/mTOR, and Ras downstream pathways	Apoptosis Autophagy To a less extent necrosis	Mainly glioblastoma. But also several solid cancer types including pancreatic ductal adenocarcinoma, cholangiocarcinoma, multiple myeloma, ovarian carcinoma, colorectal cancer and acute myeloid leukemia.	Anticancer evidence: less available preclinical evidence, very limited clinical evidence Supportive-care evidence: less available clinical evidence.
CBC	Weak agonist at CB1 and CB2 [221] Potent agonist at TRPA1 agonist Less potent agonist at TRPV1, TRPV2 and TRPM8 [123] Intermediate agonist at TRPV3 and TRPV4 [123,220]	Elevation of circulating levels of the endocannabinoid anandamide [123]	Apoptosis To a lesser extent necrosis	Several solid tumors including breast and colorectal cancer and multiple myeloma	Anticancer evidence: less available preclinical evidence, very limited clinical evidence Supportive-care evidence: less available clinical evidence.
WIN-55,212-2 (WIN-55)	CB1 and CB2 agonist [224]	MAPK/JNK signaling leading to mitochondrial depolarization and caspase activation	Apoptosis To a much lesser extent parthanatos	Solid and liquid cancers including acute myeloid leukemia, non-Hodgkin lymphoma, myeloma, glioma, and prostate cancer	Anticancer evidence: available preclinical evidence, very limited to no clinical evidence Supportive-care evidence: very limited to no clinical evidence
JWH-133	CB2 agonist with significant selectivity over CB1 [225]	Pathways downstream CB2 such as the PI3K/Akt pathway	Apoptosis	Mainly myeloma, breast cancer (mainly TNBC), and glioma	Anticancer evidence: available preclinical evidence, very limited to no clinical evidence Supportive-care evidence: very limited to no clinical evidence
JWH-015	CB2 agonist [209]	Pathways downstream CB2 such as the ceramide/JNK pathway	Apoptosis To a much lesser extent autophagy	Mainly, mammary carcinoma, hepatocellular carcinoma, and prostate cancer	Anticancer evidence: available preclinical evidence, very limited to no clinical evidence Supportive-care evidence: very limited to no clinical evidence

**Table 3:** Comparative pharmacokinetic profiles of major phytocannabinoids in relation to major routes of administration. CBC cannabichromene; CBD cannabidiol; CBG cannabigerol; CBN cannabinol; CYP2C cytochrome P450 family 2 subfamily C; CYP2C9 cytochrome P450 family 2 subfamily C member 9; CYP2C19 cytochrome P450 family 2 subfamily C member 19; CYP3A cytochrome P450 family 3 subfamily A; CYP3A4 cytochrome P450 family 3 subfamily A member 4; CYP450 cytochrome P450; t1/2 terminal half-life; THC Δ9-tetrahydrocannabinol; Vd volume of distribution.

	Absorption	Distribution	Metabolism	Elimination/Excretion
THC	Oral Low bioavailability = 4–12% [226] Maximum blood concentration is reached at 0.66–4 h after administration [227] Inhalation, similar pharmacokinetic profile to intravenous administration- rapid and extended exposure [227] Maximum plasma concentration reached at 3–10 min [228] Bioavailability = 10–35% due to high variability across individuals [226]	Oral Vd = 4–14 L/kg [229] Inhalation Vd= 3.4 L/kg [229] Rapid brain and fat tissue uptake [227]	Oral Extensive hepatic metabolism (oxidation) THC → 11-OH-THC → 11-COOH-THC by CYP2C and CYP3A [228] Inhalation Less extensive hepatic metabolism [230]	t1/2 = 22 h (prolonged) [231] can be over 24 h in frequent users [228,231] 65% in feces and 20% in urine as hydroxylated and carboxylic metabolites [227]
CBD	Oral Maximum plasma concentration achieved after 1–4 h [232] Low bioavailability = 6% [228] Inhalation Maximum plasma concentration achieved after 3.8 ± 1.3 min [233] Bioavailability = 11–45% [226]	Vd = 32 L/kg [234] Accumulates in fat and liver [227]	Oral Extensive hepatic metabolism (oxidation) CBD → 7-OH-CBD → 7-COOH-CBD by CYP2C19, CYP3A4 [235] Inhalation Less extensive hepatic metabolism [233]	t1/2 = 1 h 5 days depending on the dose and route of administration [232] Mean t1/2 = 18–32 h [226] Hydroxylated CBD metabolite is eliminated by feces, lesser extent by urine [228]
CBN	Bioavailability through smoking = 8–77% with a mean of 41% depending on route of administration and individual metabolic profile [236]	-	Oral Hydroxylation to 11-OH-CBN [236]	Mainly excreted as metabolites in feces with minor urinary excretion [227]
CBG	Low water solubility, lipophilic [226] Oral Limited bioavailability = 6–10% [226] Maximum plasma concentrations achieved at = 1–4 h [237] Inhalation Rapid absorption and higher bioavailability = 25–35% Maximum plasma concentrations achieved at 5-10 min [226]	Extensive distribution throughout the body and in high-vascularized organs including lungs, liver, and brain. High fat tissue uptake [227] Vd= not studied, but assumed to be high [227]	Extensive hepatic metabolism by CYP450 enzymes, mainly CYP2C9 and CYP3A4 → OH-CBG, COOH-CBG [235]	t1/2 = 18–24 h, depends on dose and administration frequency [228] Metabolites excreted mainly in feces and minimally in urine [228]
CBC	Oral Low bioavailability = 6–12% [238] Inhalation Higher bioavailability = 25–45% [239] Rapid systemic absorption Peak plasma concentrations achieved within mins [239]	Highly lipophilic- high fat tissue uptake [227] Oral Rapid distribution to highly vascularized tissues: brain, liver, lungs [227] Vd = not studied, but assumed to be high [227]	Metabolized mainly by with CYP450 enzymes, mainly CYP2C9 and CYP3A4 in the liver [235]	t1/2 = 10–24 h depends on administration route and individual metabolic profile [238] Metabolites excreted mainly in feces, minimally in urine [228]

## Data Availability

No new data were created or analyzed in this study. Data sharing is not applicable to this article.
